# Review of HIV Self Testing Technologies and Promising Approaches for the Next Generation

**DOI:** 10.3390/bios13020298

**Published:** 2023-02-20

**Authors:** Amanda Bacon, Weijing Wang, Hankeun Lee, Saurabh Umrao, Prima Dewi Sinawang, Demir Akin, Kodchakorn Khemtonglang, Anqi Tan, Sabina Hirshfield, Utkan Demirci, Xing Wang, Brian T. Cunningham

**Affiliations:** 1 Department of Bioengineering, University of Illinois at Urbana-Champaign, Urbana, IL 61801, USA; 2 Nick Holonyak Jr. Micro and Nanotechnology Laboratory, University of Illinois at Urbana-Champaign, Urbana, IL 61801, USA; 3 Department of Electrical and Computer Engineering, University of Illinois at Urbana-Champaign, Urbana, IL 61801, USA; 4 Center for Genomic Diagnostics, Woese Institute for Genomic Biology, Urbana, IL 61801, USA; 5 Center at Stanford for Cancer Early Detection, Department of Radiology, School of Medicine, Stanford University, Palo Alto, CA 94304, USA; 6 Department of Chemical Engineering, Stanford University, Stanford, CA 94305, USA; 7 Center for Cancer Nanotechnology Excellence for Translational Diagnostics (CCNE-TD), School of Medicine, Stanford University, Stanford, CA 94305, USA; 8 Special Treatment and Research (STAR) Program, Department of Medicine, SUNY Downstate Health Sciences University, Brooklyn, New York, NY 11203, USA; 9 Department of Chemistry, University of Illinois at Urbana-Champaign, Urbana, IL 61801, USA

**Keywords:** HIV self-testing, HIV viral load, viral testing, biosensor, LFA

## Abstract

The ability to self-test for HIV is vital to preventing transmission, particularly when used in concert with HIV biomedical prevention modalities, such as pre-exposure prophylaxis (PrEP). In this paper, we review recent developments in HIV self-testing and self-sampling methods, and the potential future impact of novel materials and methods that emerged through efforts to develop more effective point-of-care (POC) SARS-CoV-2 diagnostics. We address the gaps in existing HIV self-testing technologies, where improvements in test sensitivity, sample-to-answer time, simplicity, and cost are needed to enhance diagnostic accuracy and widespread accessibility. We discuss potential paths toward the next generation of HIV self-testing through sample collection materials, biosensing assay techniques, and miniaturized instrumentation. We discuss the implications for other applications, such as self-monitoring of HIV viral load and other infectious diseases.

## 1. Introduction

With increased longevity and multi-morbidities, in-clinic visits will become a growing barrier to healthcare as the population ages [1]. Self-testing may provide healthcare systems with a more feasible way to engage and monitor patients using telehealth services that provide easier access, reduced cost, and improved adherence to therapeutic and preventive regimens [2,3]. Additionally, self-testing may provide a healthcare pathway to subgroups historically discriminated against or less reachable by the healthcare system, including sexual and gender minority populations, people who use drugs, and rural populations.

Self-testing, defined as a diagnostic test in which a person provides their specimen and performs the test themselves, is a potential alternative to conventional in-person specimen collection at a clinic, followed by diagnostic tests performed in a laboratory. Self-sampling is an intermediate approach in which a patient collects a sample themselves using a device that facilitates safe storage and shipping to a laboratory. Self-sampling can minimize the need for in-person clinic visits but is associated with lengthy delays for learning the diagnostic result. Self-testing is feasible when self-sampling devices are combined with accurate and simple at-home diagnostic assays. Importantly, self-testing offers the potential to expedite and simplify the test-to-result process, reach a wider patient population, provide greater privacy, and utilize a low specimen volume. When self-tests are combined with internet connectivity and personal mobile devices, they can integrate with telehealth services to improve monitoring and adherence to medication, and to inform the patient by video or telephone about how to modify their health behaviors [4,5]. In the context of human immune deficiency virus (HIV) viral load monitoring, self-testing offers the potential to inform patients about their viral load status (for example, virally suppressed or unsuppressed) [6]. In the future, HIV viral load self-testing could be used to determine if a person receiving antiretroviral therapy (ART) should maintain their current regimen, or to determine if the treatment should be modified, for example, by evidence of a drug-resistant strain.

The Centers for Disease Control (CDC) recommends HIV screening as part of general routine healthcare for everyone between 13 and 64 years old regardless of risk. For those in higher risk groups, the CDC recommends getting tested at least once a year [7]. The World Health Organization (WHO) recommends that three consecutive positive tests are needed for a conclusive HIV diagnosis for underdeveloped countries, which is expected to further drive the need for inexpensive and accurate self-testing and self-sampling approaches [8].

It is important to note that there is no current test that can immediately detect HIV infection. Instead, all tests require a time window between exposure and detection [2]. Within this window period, an individual with newly acquired HIV can unknowingly spread the disease to others. Thus, early detection of HIV enables individuals to obtain medical treatment early to reduce adverse health effects [9]. The early detection window has decreased through advancements in laboratory-based tests with lower limits of detection of HIV antigens in blood or oral fluid. Third and fourth generation HIV tests use synthetic HIV1,2 IgG and IgM antibodies to detect p24 antigens. Most third-generation tests have a window period of approximately 22 days after initial infection [2].

Following a preliminary positive test result from a self-test, the current clinical workflow for laboratory-based HIV diagnosis is to initially utilize a 4th generation HIV immunoassay that can detect both HIV antibodies and p24 antigen. If a positive indication is obtained, a differentiation immunoassay is performed next to determine the strain of HIV, as treatment plans are different for HIV-1 and HIV-2 [10]. Currently, there is only one approved differentiation immunoassay that is approved by the Food and Drug Administration (FDA), Geenius HIV1/2 from Bio-Rad [11]. If the differentiation immunoassay is indeterminate, or if an early infection is possible, a HIV-1 nucleic acid test is also performed. This testing algorithm has been recommended by the CDC since 2014, replacing HIV-1 immunofluorescence and western blot assays [12]. After diagnosis, viral load and T cell counts will be tested pretreatment, 4–6 weeks after treatment begins, and every 3–6 months thereafter [13].

The COVID-19 pandemic expedited self-testing technologies like no other time in history. The global crisis that rapidly unfolded in early 2020 spurred the need to reduce morbidity and mortality in the general population due to SARS-CoV-2, a highly infectious respiratory virus that is spread through aerosols in exhaled breath. Unprecedented international research and development funding, global scientific collaboration, and intense commercial competition led to the development of self-testing kits for SARS-CoV-2 to help contain the spread of infection and signal the need for healthcare visits or hospitalization. In the three years since the onset of COVID-19, several SARS-CoV-2 self-tests have been successfully brought to market, while many exciting advances were reported in the scientific literature that have yet to develop into commercial products. In the United States, self-tests were available online at no cost to U.S. residents [14], with the goal of improving access to self-testing and self-regulation of COVID-related health behaviors, such as mask-wearing and social distancing. Despite many important achievements in sample pre-processing, detection methods, biochemistry methods, sensors, and instrumentation for rapid and simple SARS-CoV-2 testing in viral transport media, saliva, and exhaled breath, the predominant methods utilized during the pandemic for self-testing and point-of-care testing relied upon established pre-existing technology such as lateral flow test strips and polymerase chain reaction (PCR) [15]. While the COVID-19 pandemic is not yet concluded, many of the diagnostic technologies developed in the past three years are poised to make an impact for other pathogen-driven diseases where reduced cost, reduced sample-to-answer time, and simple workflows can improve health outcomes.

The fast-paced technology development for self-testing diagnostics surrounding COVID-19 stands in contrast to the historically slow progress of these systems to address the longstanding HIV epidemic. Because HIV continues to be clustered in men who have sex with men (MSM) [16], and transmission is fluid-based and largely sexual in nature [17], progress in HIV self-testing technologies has been hindered by politics, racism, discrimination, and stigma [18]. Despite historical setbacks, HIV treatment for people living with HIV has made tremendous strides in the past few decades to the point where HIV is considered a chronic illness [19]. Additionally, with the 2012 FDA approval of Truvada™ [20], HIV medication taken to prevent transmission of HIV in negative persons has been highly effective, especially among MSM populations; however, cost barriers have resulted in significantly lower uptake among Black and Hispanic/Latino MSM than White MSM [21].

**Figure 1:**
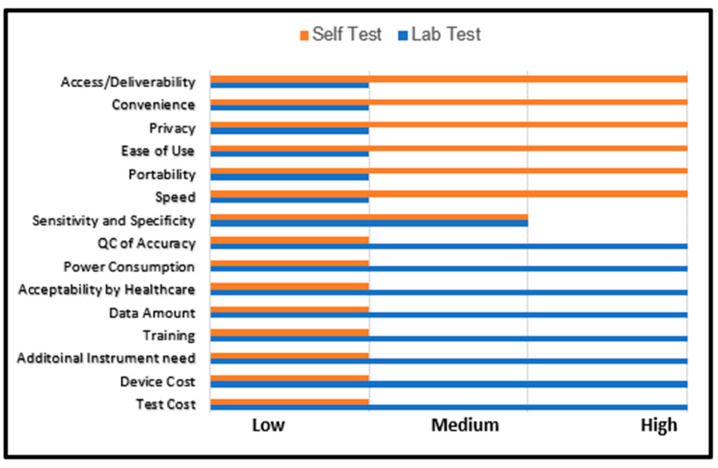
Summary of the differential characteristics between diagnostic tests performed in laboratory environments compared to self-test diagnostics.

A key challenge to reducing the spread of infectious diseases and managing their treatment is regular testing. While HIV self-testing technologies have been slow to advance, HIV biomedical prevention modalities are now very effective, when used in conjunction with frequent testing. The uptake of HIV self-testing has been impeded by structural barriers, including stigma and the low investment in research and development relative to other chronic conditions such as high cholesterol and glucose [22]. When an infectious disease impacts only certain groups, the stigma associated with receiving a diagnosis may prevent individuals from seeking laboratory-based testing offered in conventional clinic environments. Self-testing is one approach to addressing stigma [23]. HIV self-testing may be noninvasively performed with oral fluid and, more recently, with whole blood [24]. Figure 1 displays the differences between self-testing and laboratory testing. As self-testing excels at privacy and ease of use, it could provide a stigma free testing alternative while providing similar sensitivity and selectivity as laboratory testing. Like the SARS-CoV-2 self-tests, the sensitivity and specificity of HIV self-tests have limitations. Unlike the SARS-CoV-2 self-tests, the unsubsidized cost of HIV self-testing (for example, OraQuick™, ~$40 per test [25]) leaves HIV self-testing inaccessible. Thus, there is currently an unmet need to develop rapid, accurate, inexpensive, and simple methods for HIV self-testing—both in the contexts of HIV diagnosis and (future) viral load self-monitoring.

In this paper, we highlight recent advances in HIV self-testing, focused upon technological developments derived from novel materials, particularly those that we expect to make a positive impact on HIV. We begin by reviewing the current state of HIV diagnosis self-testing by nucleic acid tests and antigen tests in light of recommendations by the CDC for sexually active HIV-negative people, including those considered at higher risk for HIV (e.g., people who inject drugs, MSM, transgender women). While there are currently no self-tests for HIV viral load approved by the FDA, we discuss the requirements that are currently met with laboratory-based methods to develop such a test. Next, we briefly review commercially available self-sampling methods, leading to a discussion of the sample preparation methods for self-testing, and a review of promising sample preparation methods for self-testing in the research literature. Through a review of SARS-CoV-2 self-test technologies, we highlight newly developed assay technologies for detection of virus-specific nucleic acid sequences, antigens, antibodies, and intact viruses. While SARS-CoV-2 tests are generally performed upon viral transport media from nasal swabs or from saliva, we extend our thinking toward how these methods can be adapted for detection of HIV in blood and oral fluid. We review not only the assay methods in terms of their simplicity, robustness, and capability for quantitative accuracy, but also the instrumentation used for self-tests in terms of their cost, size, and ease of use. We conclude with a discussion of the remaining hurdles for more widespread adoption of HIV self-testing and offer a future outlook for the opportunities for novel materials and methods to provide solutions.

## 2. Current State of HIV Self-Testing

In 2014, the Joint United Nations Programme on HIV and AIDS (UNAIDS) set a “95-95-95” goal, in which, by 2030, 95% of individuals living with HIV will be diagnosed, of whom 95% will be on ART, and 95% will achieve sustained virologic suppression. Currently, 41 HIV detection products have been pre-qualified by the WHO [26]. Of the 41 WHO pre-qualified in-vitro diagnostic (IVD) products, 27 are intended for rapid HIV diagnostic testing by healthcare professionals in resource-limited and point of care (POC) settings, while six products are intended for lay users to perform self-testing without the help from trained personnel. Though not pre-qualified by WHO, six additional products are also currently available, some of which have obtained regulatory approvals or registration from other institutions, such as the U.S. FDA or Conformité Européenne (CE).

Due to their simplicity and low cost, lateral flow assay (LFA)-based diagnostic devices have been widely adopted for diagnostic scenarios where rapid results are required [27]. LFA-based tests can be performed on a variety of biological samples, including sweat, saliva, plasma, serum, and whole blood. The testing does not require expensive laboratory equipment and the results are available in 5–30 min. Moreover, sample quantity required for detection is less than needed for conventional confirmatory HIV diagnostic assays, such as enzyme linked immunoassay (ELISA), thereby allowing affordable, sensitive, specific, user friendly, rapid, equipment free, and deliverable (ASSURED) detection of HIV infections.

Over-the-counter (OTC) LFA self-testing kits rule the market with their low manufacturing cost, simple design, low sample volume, and user-friendly visual format. LFAs are paper-based devices that require a small fluid sample (e.g., saliva, urine, blood). Once the fluid sample is applied to the test, a sample buffer carries the components of the fluid through a reaction membrane. Part of the sample buffer includes a labeling probe. A common labeling probe used in HIV LFA self-tests is gold nanoparticles (AuNPs) because they bond well with biomolecules and can be visually identified. For HIV self-tests, the labels will bond with the desired molecule (e.g., p24 antigen) and then further migrate to the detection pad. Within the detection are two test result areas, indicating a standard control and test line. For example, two lines indicate a positive test result, one line would indicate a negative result, and no lines would indicate an invalid result. Of note, the stationary detection molecules are located where the result lines appear on the test. For HIV testing, p24 antibody are captured by the p24 antigen detection molecules.

**Figure 2:**
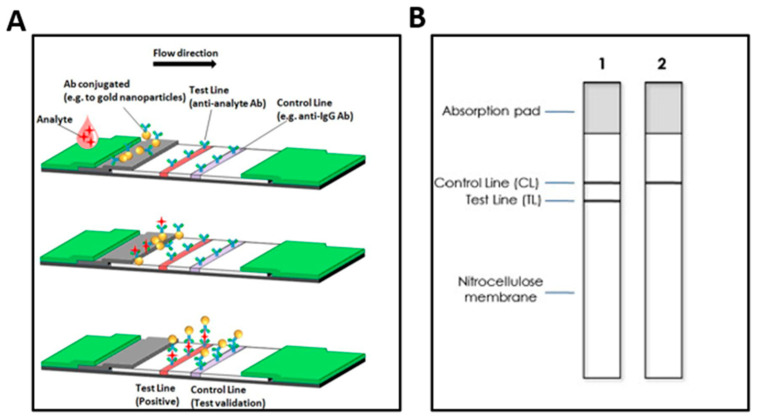
(**A**) Schematic representation of LFA mechanism. At the top: the sample is deposited on the sample pad and migrates towards the reaction membrane. Middle: the labeling probes bind the target analyte and (bottom) complex migrates to the test line, where the probe bound target is captured [28]. (**B**) Schematic representation of (1): positive and (2): negative outcomes [29]. Not shown are invalid outcomes.

To create the test lines, capture molecules (antigens or antibodies) are immobilized to react with the biomolecular complexes, forming a structure where the analyte of interest is sandwiched between the capture and label molecules. The control line is constructed by immobilizing with secondary capture molecules that react with the unbound, label molecules to indicate proper liquid flow and validity of the assay. HIV detection has several options for desired analyte and capture molecules. To detect HIV-1/2 antibodies, recombinant envelope glycoproteins (GPs) are conjugated to the labeling probes; GP41, GP120, and GP160 for HIV-1 antibodies and GP36 for HIV-2 antibodies. The HIV antigen (p24 nucleocapsid protein) is detected by conjugating monoclonal anti-p24 antibodies to the labeling probes. The working principle of the LFA is illustrated in Figure 2.

LFA technologies for early HIV detection have advanced in the past decade through novel nanomaterials that enabled reduction of detection limits for the targeted analyte through greater contrast during visualization. Beyond forming stable bonds with biomolecules and being visible to the naked eye, AuNPs with diameters in the 10–100 nm range can absorb and scatter light at specific wavelengths in the visually observable portion of the spectrum through localized surface plasmon resonance (LSPR), which can amplify the amount of visual light that is scattered, increasing the signal from these labelling probes. Utilizing AuNPs as the foundation, many approaches to increase signal have been demonstrated. For example, Chen et al. used small and large AuNPs to amplify the signal through the formation of gold clusters [30]. Yang et al. deposited silver on AuNPs to enhance sensitivity by 100-fold [31], and Panferov et al. applied a gold enhancement technique to increase the size of the AuNPs post-reaction to further improve the assay sensitivity [32]. Alternative nanomaterials have also been explored and applied as labeling probes. For example, a rapid testing device utilizing colloidal selenium to detect HIV-1/2 antibodies present in whole blood, serum, or plasma (Determine™ by Abbott Laboratories) demonstrated sensitivity of 100% and specificity of 98.93% when tested on a panel of 1079 samples [33]. Colloidal selenium tags offer potential advantages compared to AuNP in terms of cost and manufacturability [34]. Though not yet used in HIV detection, colloidal carbon has also demonstrated excellent potential as an LFA labeling probe due to its high stability, bio-compatibility, and ease of fabrication, while offering high signal-to-noise ratio due to the contrast between the white LFA membrane and the black tags [35].

Quantitative analysis of LFA with portable or laboratory-based readout instruments has been developed. Fluorescent dyes, paramagnetic particles [36], and quantum dots [37] represent additional categories of nanomaterial tags that have been utilized in LFA that are generally detected with an instrument rather than by visual observation. Tag-free readout of an LFA has been demonstrated using Surface-Enhanced Raman Scattering (SERS)-based assays in which the probe-target biomolecular complex could be detected through its spectroscopic scattered signature [38]. In the context of HIV self-testing, instrument-based detection is an obstacle for adoption, unless the instrument cost can be in the $20–500 range.

## 3. Current State of Self-Sampling

A summary of currently available HIV self-tests, usable by the layperson to clinician to still in research development is seen in Table 1. Most self-tests are LFA style, performed with finger-pricked whole blood, except for OraQuick and Aware, which use oral fluid, while DPP HIV 1/2 Assay has the oral fluid option. While a variety of tests are available with several having FDA approval, only one is available to purchase by individuals and use at home in America. The OraQuick™ price is ~$40 per test [25]. Despite the diversity of products, all of them utilize the LFA technique. LFA-based HIV self-testing devices are expected to continue playing a significant role in reaching the UNAID goal of ending HIV by 2030. After a positive HIV diagnosis from a self-test, CDC recommends conducting additional confirmatory tests [10], requiring an in-person visit to a testing facility, which may not be desirable for those who wish to be anonymous, or feasible for those living in remote areas. However, the importance of confirmatory testing was emphasized by a recent study [39] in which three HIV rapid tests, including Determine™ HIV-1/2, Uni-gold™ HIV-1/2, and StatPak™ HIV-1/2, were performed on more than 200,000 participants, in which, reviewed results of a testing algorithm using three HIV confirmatory tests to reduce false positive rates. Utilizing laboratory-based testing as a gold standard, a positive predictive value (PPV) of 94.5% was obtained for self-tests. A separate study to evaluate the accuracy of oral self-test kits for identifying people with undiagnosed HIV in Ethiopia (OraQuick™, Determine™, and StatPak™) revealed a false negative rate of 0.5% and a PPV of 100% [40]. Self-sampling combined with laboratory-based testing offers a path that enables individuals with positive HIV self-tests to utilize the rapid test as an initial screen, while utilizing the services of a more sensitive laboratory-based test without making an in-person visit to a clinic. Self-sampling methods via lance or fingerstick is currently one of the ways that separates tests from being done by untrained individuals or by clinicians, as noted in Table 1 by asterisks.

Since its introduction in the 1860s, dried blood spots (DBS) have been used as a method for collection, preservation, and shipment of blood samples for qualitative or semiqualitative diagnosis [79]. Diagnostic applications of DBS include newborn screening, viral load determination, and serology testing. DBS can be an inexpensive solution for disease detection especially in areas of minimal infrastructure, as DBS reduces the logistics for collecting, preserving, and transporting blood specimens. [80]. The simple procedure begins with application of a fingerpick-derived droplet of blood on filter paper, followed by drying in an open environment at an ambient temperature, and transport in a sealed bag with desiccant. DBS samples may be stored for extended time periods in either a refrigerated or frozen state. DBS sampling was adopted for HIV-1 diagnosis in 1996 through the FDA-approved Home Access™ HIV-1 Test System. The product is an OTC kit, in which the patient performs a finger-prick on themselves, followed by mailing the sample to a laboratory, where an HIV antibody test is performed. Analysis of DBS poses analytical challenges, such as variation of blood content within the dried spot due to the percentage of red blood cells (hematocrit effect), contamination of the sample by the user, and insufficient sample volume.

To address these challenges, volumetric absorptive micro-sampling (VAMS) was developed and several devices that incorporate this technique are commercially available, including Mitra™ (Neoteryx, Torrance, CA, USA), Capitainer qDBS™, HemaPEN™ (Trajan Scientific and Medical, Victoria, Australia), and the HemaXis™ DB10 (DBS system SA, Gland, Switzerland). Mitra™, for example, consists of a hydrophilic polymer tip that wicks a precise volume of fluid by capillary action. Specific sample volumes can be collected (10, 20, or 30 μL) through products that are engineered with different tip sizes [81]. Capitainer™ qDBS is a micro-sampling tool that enables the collection of two fixed volume (10 μL) DBS using microchannels and capillary force. Neoteryx claims that the device allows a precise collection of the blood sample irrespective of the hematocrit effect, which is supported by a number of literature reports [82]. HemaPEN™ uses 2.74 μL capillaries coated with EDTA (an anticoagulant) to collect blood on filter paper media within approximately 10 s until all the capillaries are filled and the dried samples are retrieved by opening the device with a supplementary opening tool [81]. HemaXis™ DB 10 contains a conventional DBS filter paper and four capillary channels with inlets for direct contact with a blood drop and outlets to indicate when a sufficient volume of blood is collected. Once the capillaries are filled, the DBS filter paper touches the outlets by the user manually closing the device and the accurate volume of blood (10 μL) is transferred to create DBS [83]. HemaSpot™ HF (SpotOn Sciences, San Francisco, CA, USA) has eight identical blade-shaped filter paper pieces arranged in a spiral to collect 9–10 μL of blood per blade. After blood collection, a desiccant within the cartridge rapidly dries the blood to preserve it for transit to a lab, which can occur at ambient temperature. The cartridge is put inside a bag and sent to a laboratory for testing.

While the DBS-based VAMS devices collect relatively small volumes, devices that collect larger volumes of liquid whole blood (>100 μL) have recently become available. These include the TASSO-SST™ (Tasso Inc, Seattle, WA, USA) and OneDraw™ (DrawBridge Health, San Diego, CA, USA). Designed to be placed on the upper arm, the TASSO-SST device collects 200–300 μL of liquid whole blood by pressing a button, which initiates puncturing of the skin with an integrated lancet, followed by withdrawal of blood into capillary tubes using vacuum pressure within 5 min. Using similar technology, the TASSO-M20 gathers four dried samples of 17.5 μL volume each. The OneDraw™, when attached to the upper arm, draws a liquid whole blood sample (~150 μL) with a push of a button. The device comes with a removable cartridge comprised of filter paper impregnated with blood stabilizing reagents for transport and storage.

In addition to blood, self-sampled oral fluid (mucosal transudate) is widely used as a method for HIV diagnosis. An example product is the OraSure™ HIV-1 Oral specimen collection device. Oral fluid can be easily sampled in a non-invasive way by gently swabbing the gums with a polyester or rayon-tipped stick, followed by insertion of the tip into transport media in a sealable tube for transport. The swab material can absorb substantial sample volume, and the composition of the material can be engineered to achieve greater efficiency of release volume. Polyester and Rayon fiber swabs material can absorb significant amounts of sample volume but have minimal release volume (~33%) [84,85], while Nylon flocked swabs elute 97% of the sample off the swab very quickly with a ~5-s vortex [83]. Swab collection devices are considered Class 1 devices by the FDA, and, thus, stringent requirements must be met for their composition and performance. Currently, there are ten self-sampling products available, summarized in Table 2, of which two utilize oral fluid, and eight require whole blood in either wet or dried state.

## 4. HIV and CD4+ T-Cell Separation, Capture, and Detection

**Figure 3:**
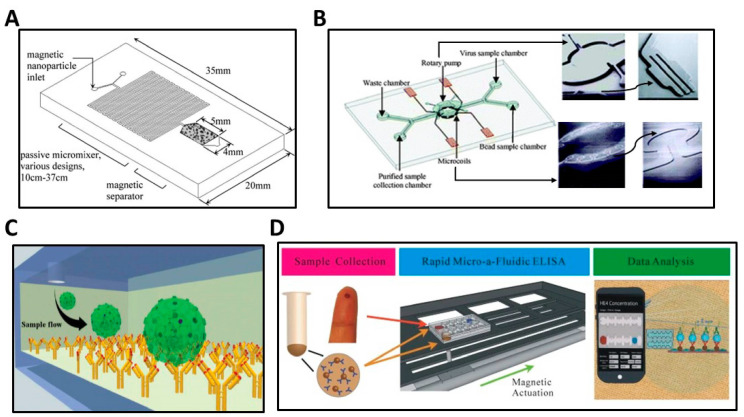
(**A**) Schematic of a microfluidic device that combines superparamagnetic nanoparticles to isolate and concentrate HIV-1 from plasma. Plasma samples were mixed with antibody-functionalized nanoparticles, and then the mixture was trapped in the magnetic separator chamber [86]. Reprinted with permission from Anal Chem. Copyright 2009 American Chemical Society. (**B**) Schematic of a microfluidic chip integrating a micromixer and a bead collection separator into a single chip to isolate and enrich Dengue Virus [87]. (**C**) Microchannels were fabricated to capture HIV particles using immobilized anti-gp120 antibody on a glass substrate. The capture efficiency of HIV subtypes A, B, and C was then characterized by RT-qPCR [88]. (**D**) Workflow of the m-ELISA platform, which provides CD4+ cell count based on a colorimetric readout on a cell phone for monitoring ART in people living with HIV. Blood samples and antibody-functionalized magnetic beads were loaded into the chip, which was then placed on a permanent magnet. Automation was performed using a software program to control the stage and complete the entire ELISA process. Lastly, the chip colorimetric readout was imaged using a cell phone, and the analysis was performed using an integrated mobile application [92]. Images reused under CC BY-NC-ND 3.0 License (https://creativecommons.org/licenses/by-nc-nd/3.0/) accessed on 5 December 2022.

The low concentration of HIV in clinical samples poses a considerable challenge in developing low cost, point-of-care viral assays. Separating virus from blood for self-testing can be used to pre-concentrate virus from blood into a smaller volume to enhance detection limits. Microfluidic systems can offer separation and analysis of the capture virus. For example, one study (Figure 3A) combined superparamagnetic nanoparticles in microfluidics to purify and concentrate HIV-1 from plasma, and showed 40–80 fold enrichment of target viruses [86]. Further analysis was done to extract the nucleic acid by membrane lysis on the same chip. A similar approach (Figure 3B) showed capture of dengue virus postulating 20-fold enrichment from 1 mL starting sample, assuming 100% efficiency [87].

Another study used a microfluidic chip to capture multiple HIV subtypes (A, B, and C) using protein G-based anti-gp120 antibody immobilization (Figure 3C), where the different HIV subtypes were derived from viral culture supernatant and spiked in whole blood. The capture efficiency was 75.73% for subtype A, 73.67% for subtype B, and 74.67% for subtype C across 103–105 viral copies/mL [88].

As an alternative to counting HIV viral load, CD4+ T lymphocyte count can be used to monitor HIV ART [89]. A decreasing number of CD4+ T lymphocytes can be a marker for the host’s compromised system [90]. One of the challenges in assays for viral load and CD4+ cell count is the cost and complexity, which poses a barrier for self-testing scenarios [89]. For example, the precise fluid flow in the microfluidic design is a critical barrier for diagnosis at the point of care, especially for automation in self-testing [91]. Combining a microfluidic system with ELISA readout offers a route toward more rapid measurement of CD4+ cell count (Figure 3D). A study using micro-a-fluidic ELISA (m-ELISA) counted CD4+ cells in an automated manner with the results shown on a cell phone [92]. The limit of detection (LOD) of m-ELISA was 30 CD4+ T lymphocytes in a 9-min reaction. Moreover, it showed 97% accuracy at the clinical cutoff of 350 cells/µL as recommended by WHO in 2013 [93].

## 5. Self-Testing Technology Advances in Research and Development

In the context of HIV diagnosis, the target analyte for detection may be the presence of virus-specific nucleic acid sequence, a viral antigen, or the intact virus. Detection of HIV antibody can be utilized to indirectly detect infection via activation of the immune system. Here, we summarize several recent reports in the research literature that demonstrate promising combinations of low limits of detection for one of the analyte types, while also offering workflows and readout methods that may become compatible with the challenging demands of self-testing.

### 5.1. Antigen Tests

HIV p24 antigen can be detected in circulating blood within a few days post-infection and is detectable sooner than HIV antibodies, whose presence and concentration is dependent upon the response of the infected person’s immune system [94]. After HIV exposure, on average, it takes approximately 13 days for the immune system to produce a detectable HIV antibodies concentration [95]. Due to the potential for post-exposure detection within a shorter window, p24 antigen has been utilized as the most prevalent HIV detection biomarker for initial diagnosis in laboratory-based tests [96]. Therefore, p24 antigen is also a common biomarker target for newly developed POC and self-testing technologies intended for early initial diagnosis. Sensing transduction methods that include fluorometric [97,98], colorimetric [99], and magneto-nano biosensors [100,101] have been reported recently.

**Figure 4:**
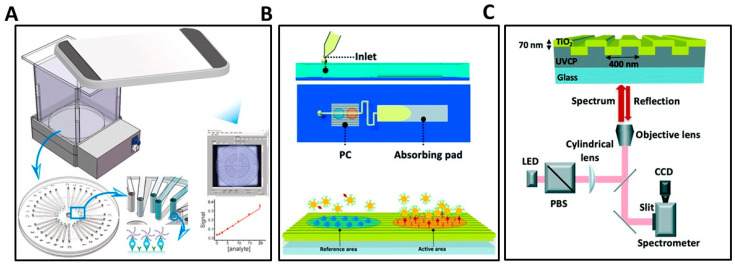
(**A**) Illustration of the microchannel-based HIV p24 antigen assay using colorimetric detection of a sandwich assay that generates a visually observable product through an enzyme-substrate interaction with a labeled detection antibody. Schematic diagram of the three-layer microchannel device where the blue visible product was captured by smartphone and determined the concentration of HIV-1 p24 antigen [106]. (**B**,**C**) Illustration of microfluidic HIV p24 antigen detection utilizing a photonic crystal and sandwich immunoassay [102]. (**B**) The sample enters the inlet area and introduces to PC by absorbing pad. (**C**) Illustration of captured antibody and blocking proteins on active and reference areas of the PC biosensor and schematic drawing of the PRAM where reflected light intensity from a red LED is observed and processed to quantify HIV-1 p24 antigen.

For example, Li et al. reported a POC p24 antigen detection approach utilizing a triple-layer microchannel array chip combined with smartphone-based readout (Figure 4A). To improve the sensitivity and simplicity of the colorimetric microchannel immunoassay, a three-layer microchip was developed along with the red, green, and blue (RGB) smartphone detection cassette. An HIV p24 monoclonal antibody served as the capture antibody and a biotinylated polyclonal antibody was utilized as a detection antibody. This sandwich immunoassay generates a signal in the presence of p24 through a tag comprised of streptavidin with a horseradish peroxidase (HRP) label, which generates blue-colored liquid in the presence of a substrate. Detection takes place through imaging of the microchannels. The smartphone records an image of the cartridge while it is inserted into the detection instrument that illuminates the cartridge with a light emitting diode (LED) while screening out ambient light. The observed color showed a strong linear correlation with the HIV p24 concentration in human serum from 0–1.25 ng/mL (R^2^ = 0.9284) with a LOD of 20 pg/mL regardless of the presence of other proteins [99]. This small, low-cost, battery-powered colorimetric microchip integrated with smartphone data processing demonstrated potential for simple and low concentration HIV p24 detection with potential to be developed into a self-test.

In another recent example, Che et al. demonstrated an assay approach called Activate Capture + Digital Counting (AC + DC) for quantification of HIV p24 in human serum utilizing a self-powered microfluidic cartridge and a photonic crystals (PC) biosensor [102]. In Figure 4B, the sample was mixed with antibody-functionalized gold nanoparticles (AuNPs) that are drawn into a microfluidic channel with an absorbing paper pad. Detection occurs in a single step, with no washing, as the p24 is captured with an immobilized antibody, and then labeled with an AuNP functionalized with the detection antibody, forming a sandwich complex. Each captured p24 molecule is labeled with one AuNP, which are subsequently digitally counted by a biosensor microscopy approach called Photonic Resonator Absorption Microscopy (PRAM) [17,103,104,105]. The assay includes the active area for selective capture of p24 and a reference region prepared with a blocking protein (Figure 4B). The antibody conjugated AuNPs bind to the target molecules in the test samples and then captured on the PC surface. The AuNPs are selected to have an LSPR wavelength that matches the PC biosensor resonant reflection wavelength, enabling PRAM to observe them with high signal-to-noise ratio. The AuNPs quench the resonant reflection to generate a dark spot on an image sensor when light from a red LED illuminates the PC from below (Figure 4C). This study successfully demonstrates HIV-1 p24 antigen detection from a 40 μ spiked-in human serum sample with a dynamic range of 1–103 pg/mL within a 35 min process, suggesting the capability of implementing an ultrasensitive and fast point-of-care detection [102].

### 5.2. Antibody Tests

The human body starts producing antibodies within days of HIV infection. However, currently available laboratory-based HIV antibody tests can only identify that an infection has occurred 23 to 90 days after exposure. Meanwhile, nucleic acid tests can only detect the presence of HIV approximately 33 days after exposure as the virus infects cells, replicates, and increases its viral load [107]. The presence of detectable antibody can indicate both recent or prior infections, while nucleic acid testing represents the presence of an active infection [108]. Since the first generation of HIV diagnostic assays in 1985, subsequent generations, all focused upon detection of HIV antibodies, have introduced new approaches and capabilities. Most FDA-approved HIV rapid tests are antibody tests, for example, OraQuick^®^ Advance Rapid HIV-1/2 Antibody test, Reveal™ G-2 Rapid HIV-1 Antibody test, and Uni-Gold Recombigen^®^ HIV test. While immunoglobulin G (IgG) antibodies are the target analytes in the first through fifth generation HIV diagnostic assays, immunoglobulin M (IgM) was introduced in the third generation [109]. Antibodies detected with laboratory-based technologies have been proven effective as reliable biomarkers due to their high sensitivity and specificity [110].

**Figure 5:**
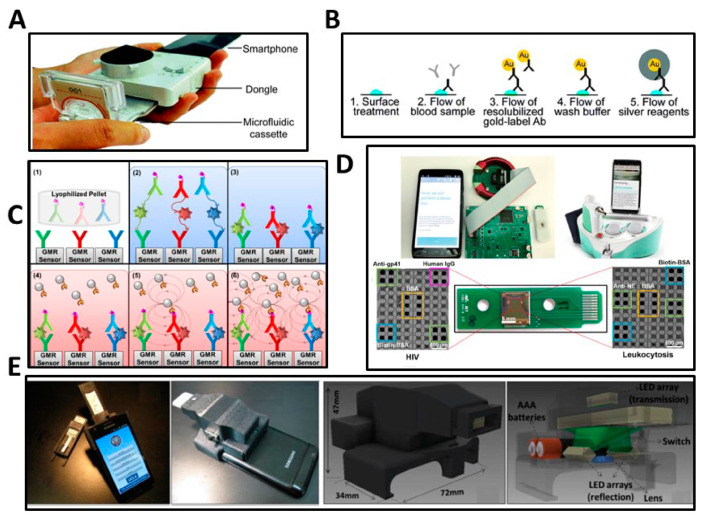
(**A**) Image of the smartphone dongle for an HIV antibody self-test [113]. (**B**) Schematic of the immunoassay workflow: (1) Test zone is functionalized with HIV antigens gp41 and gp36. (2) Flow of the blood sample allows binding of the antibody to the surface-coated antigen. (3) Flow of gold-labeled secondary antibodies. (4) Wash buffer removes the unbound antibodies. (5) Flow of the silver reagent with reducing agents to create an optically darkened zone [113]. (**C**) Schematic of wash-free GMR-based immunoassay workflow: (1) GMR sensors are functionalized with different capture molecules. (2) Test samples are added to the sensor and the target of interest is captured and detected by biotin-labeled detection probes. (3) Sandwich immuno-structures are formed on the sensor surface. (4) Streptavidin-coated MNPs are added and bind to detection probes. (5) Bound MNPs’ local magnetic field will change the sensor resistance, generating an electrical signal correlated with the analyte concentration. (6) MNPs are added again to enable higher signals [100]. (**D**) Image of the smartphone-based self-testing platform and the GMR nanosensor chip and circuit board with functionalized sensor array for HIV detection. Eight sensors are functionalized with Anti-gp41 capture antibody along with positive controls Biotin-BSA and Human IgG, and BSA as a negative control [100]. (**E**) Different views of the smart RDT reader connected to a smartphone and renderings of the optical reader. The RDT reader utilizes LEDs to uniformly illuminate the tests through a diffuser. Two of the LED arrays are located beneath the RDT tray and one illuminates from the top to record the reflection and transmission images [116].

Emerging POC approaches for HIV antibody testing that integrates with mobile devices (such as smartphones and tablet computers) are demonstrating the potential to complement traditional laboratory-based methods through simplification of assay protocols, portability, and low cost. The development of smartphone-based approaches has the potential to enable HIV self-testing in home settings and resource-limited areas [111]. Several research groups have combined smartphone-based detection and nanometer tags for HIV antibody detection. These nanometer-scale tags offer the ability to be designed to provide modifiable surface chemistries, high surface-area-to-volume ratios, and tunable wavelength interactions with electromagnetic fields [106,112]. For example, Guo et al. demonstrated a microfluidic-based smartphone dongle for HIV antibody self-testing from whole blood derived from a fingerstick. The 3D-printed smartphone accessory measures the immunoassay using gold-labeled secondary antibodies with silver amplification (Figure 5A). The test utilizes single-use disposable microfluidic cassettes that are functionalized with HIV antigens gp41 and gp36, followed by 2 μL of the lysed blood sample drawn through the microfluidic cassette. HIV antibodies in the blood sample are selectively captured by the antigens, which subsequently allow gold-labeled secondary antibodies to form a sandwich structure. Subsequently, silver nitrate and reducing agents are drawn from the cassette to initiate the growth of a silver precipitate onto the gold particles, which generates an optically dark zone. The assay readout and diagnostic result is determined through illumination of the assay region with low-power LEDs and detection of gold-labeled secondary antibodies with silver amplification through photodiode illumination (Figure 5B). The assay procedure takes 15 min and provides results with 95% sensitivity and 95% specificity with the ability to perform multiplexed viral pathogen testing of additional sexually transmitted disease such as syphilis [113,114].

In a further recent example, Ng et al. demonstrated a smartphone-based self-testing platform that employed the giant magneto resistive (GMR) effect for HIV antibody detection in oral fluid (Figure 5D). The GMR sensor platform includes a disposable cartridge that operates by the change in the sensor’s electrical resistance due to the binding of the superparamagnetic nanoparticle (MNPs) tags in a sandwich immunoassay. The resistance change can be quantified in real-time with a circuit and correlated to HIV concentration in the sample (Figure 5C). The total assay time is 16 min with 80% accuracy for HIV antibody presence in saliva [100].

Recently, considerable effort has focused on the development of test readers to objectively and quantitatively analyze rapid antibody diagnostic tests such as LFA [115], cassette tests [113], and pads [116]. For example, Mudanyali et al. described a rapid diagnostic test (RDT) reader platform that can be used with lateral flow HIV side-stream immunochromatographic assays and other similar tests (Figure 5E). This compact (~65 g) digital RDT reader is based on a smartphone and can be imaged in reflection or transmission mode under LED illumination. The raw images are processed in real-time through a custom developed app for automatic and quantitative diagnostic result determination through analysis of the color intensities of test and control lines [115].

### 5.3. Nucleic Acid Tests

Innovations in molecular biology have led to the development of novel nucleic acid amplification technologies for efficient, sensitive, and high-throughput analyses of virus-specific base sequences [117]. Detection and quantification of low concentrations of HIV-related RNA in bodily fluids is vital for early detection, clinical care, and controlling the spread of AIDS, particularly for monitoring patients receiving ART. As ART suppresses HIV replication, patients ideally will have undetectable viral load and absence in HIV antibodies, while early detection of ART failure requires detection of the evidence of HIV resurgence at the earliest possible time, necessitating low limits of detection [118]. The current laboratory-based gold standard molecular testing method for HIV detection, reverse transcription polymerase chain reaction (RT-PCR), utilizes enzymes and thermal cycling to rapidly amplify a specific region of the viral RNA into millions of copies. The HIV RT-PCR test can detect viral RNA concentrations as low as 20–176 copies/mL [119] and as early as 10 days after exposure. However, the assay is only suitable for laboratory environments, as it requires a workflow that utilizes complex sample preparation, precise temperature control, and highly trained personnel [120,121].

In an effort to provide nucleic acid-based diagnostics that are more suitable for point of care and self-testing scenarios, several alternatives offer less stringent requirements for sample preparation and remove the need for thermal cycling equipment, while still providing enzymatic amplification of a specific RNA or DNA sequence. Approaches that have gained considerable attention include loop-mediated isothermal amplification (LAMP), recombinase polymerase amplification (RPA), nucleic acid sequence-based amplification (NASBA), Rolling Circle Amplification (RCA), helicase dependent amplification (HDA), and Strand Displacement Amplification (SDA). Compared to RT-PCR, these methods provide advantages that include simplified sample preparation, less stringent temperature control, high amplification efficiency, reduced sensitivity to amplification inhibitors, and greater tolerance for detecting a target sequence within unprocessed samples, making these assays simpler to translate to POC self-testing environments. Moreover, isothermal amplification techniques can incorporate reverse transcription, expanding the detection to RNA targets such as HIV genomes [122].

**Figure 6:**
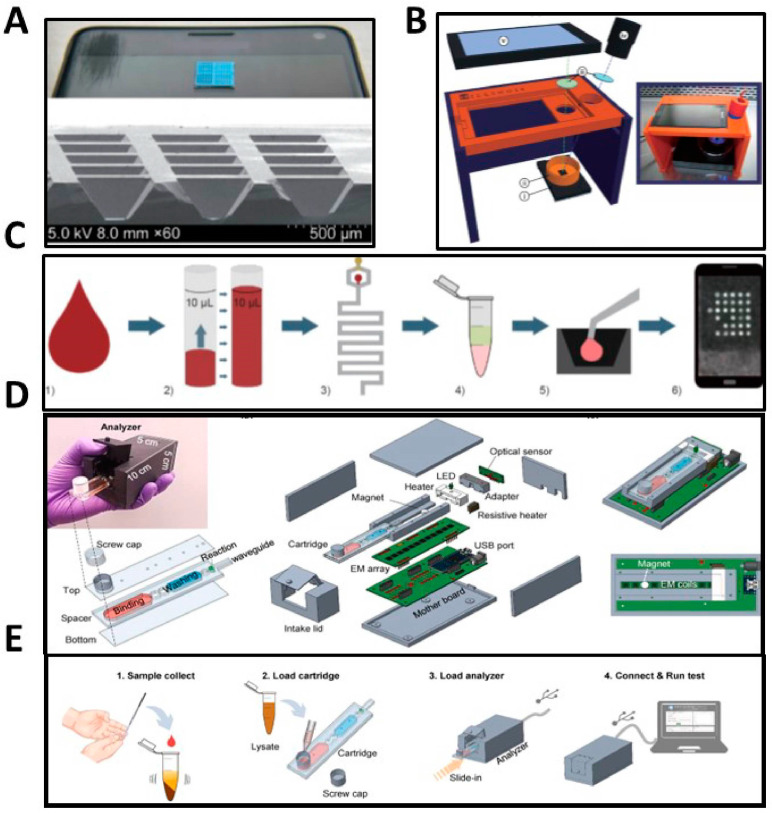
(**A**) Image of the silicon chip and detailed image of the reaction wells under scanning electron microscopy [120]. (**B**) Schematic of the smartphone-based detection apparatus from top to bottom: smartphone, blue LED, wavelength filters (blue and green), copper base with mineral oil, heating stage [120]. Images republished under CC BY 4.0 License (https://creativecommons.org/licenses/by/4.0/) accessed on 5 December 2022. (**C**) The workflow of the integrated assay process: (1) Whole blood spiked with HIV-1 virus was injected into the microfluidic apparatus where (2) 10 μL total of the blood sample was metered and then (3) the sample passed through the microfluidic lysis module. The lysis assay was added to the RT-LAMP master mix without primers (4) as the primers were dehydrated onto the silicon well where the assay was next microinjected (5). Finally, the chip was heated with a copper heating stage to 65 °C (6) and the reaction was monitored by recording images with a smartphone every 30 s [129]. (**D**) Renderings, schematics, and photo of the microfluidic with the reagents inside with the cartridge assembled to the right [125]. (**E**) Workflow of the USB HIV nucleic acid test. First, the user collects blood from a finger prick, putting 100 μL in a collection tube (1) and shakes to mix. The lysate mixture is loaded onto the cartridge (2) and sealed with the cap. The cartridge is analyzed through a USB analyzer connected to a computer (3), which then displays the results (4) [125].

LAMP is a sensitive, rapid and single-tube isothermal technique that requires a temperature of 60–65 °C. Among all the above-mentioned isothermal amplification methods, LAMP has an additional advantage, as it does not need recombinase enzyme since it does not need additional ligation steps before amplification [123]. Utilizing LAMP, detection of HIV-specific RNA was reported with a LOD of 120 copies/mL within 35 min [124]. There have been several recent efforts reported for developing RT-LAMP into HIV self-testing applications. For example, Damhorst et al. demonstrated the quantitative detection of HIV with RT-LAMP in minimally processed spiked blood samples (Figure 6A–C). The HIV spike blood was first added to a microfluidic lysis module to fully mix whole blood with the lysis buffer. The ~60 nL lysed assay droplets were next added to the master mix and transferred to a silicon chip reaction well with dehydrated LAMP primers inside, followed by the chip heating to 65 °C in a copper base with a heating stage. Detection of fluorescence generated by the reaction is performed with a smartphone apparatus containing LEDs for illumination while a smartphone records images of the chip. The assay can detect as few as three viruses in a ~60 nL droplet, corresponding to 670 viral copies/μL of whole blood [120].

Liu et al. presented a fully integrated HIV nucleic acid self-testing device of finger-pricked whole blood, which consists of a microfluidic cartridge with ready-to-use reagents inside and a compact analyzer to perform sample preparation, purification, and RT-LAMP [125]. The user controlled the process through a custom app so once 100 μL of blood was placed into the tube with premixed lysis buffer and magnetic beads, the RNA would be released into the cartridge. The device can achieve a LOD of 214 viral copies/mL in whole blood within a ~60 min sample-to-answer time (Figure 6D,E) and the authors of the study anticipate that this device can serve as a useful method for highly sensitive early-stage self-testing [125].

There are other recent reports of portable approaches for HIV nucleic acid detection that have the potential to develop into self-testing methods. For example, Myers et al. described an inexpensive, handheld, battery-powered instrument for RT-LAMP detection of the HIV-1 integrase gene in blood samples [126]. Similarly, Curtis et al. utilize heating from a chemical reaction to demonstrate HIV-1 testing from whole blood specimens using RT-LAMP [127]. Kim et al. demonstrated an enzyme-free nucleic acid amplification assay using a toehold-triggered hybridization reaction and a smartphone-based well plate fluorescence reader to detect influenza viral RNA, which could in principle be adapted to HIV self-testing [128].

### 5.4. Intact Virus Detection

The detection of intact viruses represents a novel alternative to detection of molecular biomarkers with the potential to offer simpler workflows and detection instruments that do not require viral rupture, enzymatic amplification, or temperature control [130]. Direct measurement of viral load, if sufficiently sensitive, offers a path toward initial HIV diagnosis, although it may be most useful for monitoring for the presence of free HIV in the blood due to the failure of ART [131].

Intact viruses can be detected in a label-free manner by leveraging their biophysical properties such as size, charge, weight, and refractive index [132]. A variety of electrical, optical, mechanical, and acoustic-based biosensors have been developed to convert these physical properties into signals. Label-free detection of captured virions provides real-time measurements, a simple assay protocol, and the potential for an inexpensive instrument [133,134].

**Figure 7:**
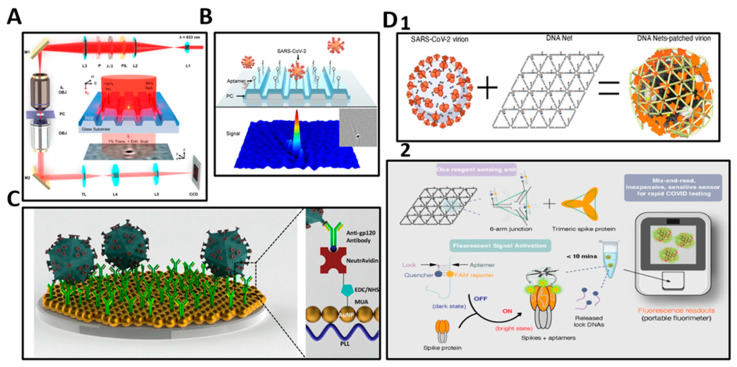
(**A**) Schematic drawing of the PRISM system for detection of intact captured viruses through interferometric scattering on a PC biosensor surface where the PC is an optically resonant substrate. Inset: Example interferometric with the PC resonance enhanced particle scattering [138]. Images republished under CC BY 4.0 License (https://creativecommons.org/licenses/by/4.0/) accessed on 5 December. (**B**) Rendering of the PC biosensor surface with example image of a SARS-CoV-2 virion with a 200 nm scale bar [135]. Reprinted with Permission from J. Am. Chem. Soc. Copyright 2021 American Chemical Society. (**C**) Schematic of nanoplasmonic viral load detection platform. Left: HIV molecules captured on the antibody-immobilized biosensing surface. Right: Rendering of the biosensor surface functionalization [136]. Reprinted with Permission from ACS Nano. Copyright 2013 American Chemical Society. (**D**) Schematic of viral capture using DDN and fluorescence reading using a compact personal fluorimeter. (1) Rationally designed a DDN based on spike-targeting aptamers that form an array of trimeric clusters and precise rhombus-shaped spike protein pattern matching. (2) The workflow of the DNA net sensor and fluorescent reading with a portable fluorimeter [137].

For example, Li et al. introduced a rapid, label-free biosensing method that through scattering of laser illumination quantifies intact viruses with single-virion resolution from human samples [135]. This PC biosensor enables amplified interferometric scattering between the forward-scattered light and the laser plane wave illumination through an approach called Photonic Resonator Interferometric Scattering Microscopy (PRISM) (Figure 7A). The PC is prepared with immobilized DNA aptamers that specifically bind with outer surface proteins of the target virus (Figure 7B). This approach achieves a LOD of 103 viral copies/mL at room temperature with a single step mix-incubate-read procedure [135].

Inci et al. presented a detection technique utilizing the immobilization of intact HIV virus specific antibodies on a plasmonic biosensor surface for capture and quantitative detection of whole blood samples due to antibody selectivity and specificity at clinically relevant concentrations (Figure 7C) [136]. Spectral analysis was analyzed and upon intact HIV virus binding, peak shifts were recorded. The platform can be used on unprocessed whole blood samples with high detection efficiency and short detection time (1 h for intact virus capture and 10 min for detection and data analysis), with detection of multiple HIV subtypes to a LOD of 98 ± 39 viral copies/mL [136].

There are further approaches for detection of intact viruses’ function by labelling the viruses with fluorescent, chemiluminescent, or nanoparticle tags that facilitate detection and quantitation. For example, Chauhan et al. presented a designer DNA nanostructure (DDN) for selective recognition and high-affinity for intact SARS-CoV-2 virion capturing (Figure 7D). DDNs are net-shaped self-assembling nucleic acid constructs with repeating units that precisely match the pattern of protein features on the virus exterior. Aptamers that specifically bind to the SARS-CoV-2 spike protein reside on each vertex of the net to precisely match the rhombus-shaped patterned spike protein. All aptamers are labeled with a fluorescent molecule that is quenched by a black hole quencher (BHQ-1) “lock” DNA that forms an aptamer-quencher duplex. Fluorescence is generated when the aptamer binds to the spike protein, resulting in a release of the quencher. The resulting fluorescence is read with a compact personal fluorimeter to give a quick SARS-CoV-2 result. This assay is simple, and inexpensive at ~$1.26 per test and had a LOD of 1000 viral genome copies/mL when tested in artificial saliva [137]. Although this method is not designed for HIV intact virus detection, it can be similarly applied to HIV detection by altering the aptamer and net formation to pattern-match an HIV external protein, such as GP-120.

## 6. Virus Self-Testing Technologies Emerging from SARS-CoV-2 Diagnostics

In this section, we review the most recent literature for various biodetection approaches that maybe suitable for HIV self-testing use through the experiential lens of rapid POC devices that emerged for the detection of SARS-CoV-2 [139]. Several recent reviews comprehensively discussed the major categories of virus detection technologies, along with their merits and disadvantages [140,141,142,143,144]; hence, these example platforms, benchtop or lab-based assays, and technologies are not covered here. In this review, we specifically focus on several new technologies or improvements to existing approaches that are ether already available OTC for at home/self-monitoring use or amenable for rapid development towards these applications.

The COVID-19 pandemic underscored the imperative need to develop diagnostic devices that can be rapidly deployed en masse to the general population to stop the spread of highly transmissible pathogens. It also highlighted the need to improve upon large-scale manufacturability, test sensitivity, sample-to-answer time, biological sample matrix (e.g., saliva, blood, nasopharyngeal fluid) independence in detection, simplicity of the entire testing process, and testing costs. Inspired by these aspects, we also propose recommendations to bring forth the next generation of HIV self-testing and viral load self-monitoring approaches, integrated devices and miniaturized instrumentation and highlight some of these as below.

As a consequence of the COVID-19 pandemic, there is a growing interest in mobile health platforms, remote patient monitoring technologies, self-monitoring tools, and portable POC systems that can also process patient samples on/in the device in an integrated fashion. For example, the single use RT-PCR device offered by Visby Inc. takes unprocessed bio samples directly and delivers a molecular readout of the presence of a target analyte [145]. A further example is the automated isothermal amplification-based molecular assay from Cue Health Inc. that incorporates a built-in small nasopharyngeal brush for sample collection that, when inserted into the device, initializes downstream RNA extraction, reverse transcription of the viral RNA to cDNA, and finally enzymatic amplification [146]. It is anticipated that these types of sample-to-answer solutions that deliver a reliable result will find expanded use in HIV detection and, more generally, in cost-effective and easy pathogen monitoring needs at-home or POC settings.

As discussed previously, the most commonly used virus detection methods for SARS-CoV-2 and HIV-1 can be classified into three overarching categories, namely (i) direct virus capture (immunofluorescence) or viral protein detection (antigen detection), (ii) indirect detection of antibodies against the viral proteins using serological approaches (ELISA and LFA) after an exposure, and (iii) detection of the viral nucleic acids by RNA/cDNA hybridization and amplification technologies such as isothermal amplification previously discussed; LAMP [147], NASBA [148], RPA [149,150], or RT-PCR amplification. Currently, all of the FDA approved in vitro diagnostics for COVID-19 detection are either antigen tests or molecular assays; for definitive confirmatory diagnosis of recent exposure or active infection is always achieved by RT-PCR. This case also holds true for detection of HIV, in that serological findings are exclusively validated by viral load testing through RT-PCR.

Various biosensing methods exist for the detection of viruses, as reviewed in detail recently [144]. These virus detection techniques can be grouped into electrical sensors (voltammetry, amperometry, field-effect transistor (FET)-based sensors, piezoresistive sensors, electromechanical sensors, and electrochemical sensors), optical sensors (fiber optic fluorescence detection, aptasensors, and colorimetric detection by the use of nanoparticles), and plasmonic methods (LSPR, Raman, and SERS). These techniques are also applicable for use in HIV viral load monitoring and self-detection.

RPA is an isothermal amplification method that uses recombinase enzymes to rapidly amplify nucleic acids without needing an annealing stage as in PCR [150]. Proviral DNA or RNA from multiple subtypes of HIV-1 has been detected via RPA in less than twenty minutes without complex equipment [149]. This RT-RPA HIV-1 assay had a LOD of 10–30 copies of HIV-1. Beyond detecting HIV-1, the assay detected 97.7% (171/175) of HIV-1 major subtypes and recombinant sequence variants, suggesting that RT-RPA application for viral RNA and proviral DNA of HIV-1 may be a highly sensitive at home testing tool for HIV diagnosis.

RT-LAMP and digital-RT-LAMP [151] are evolving low cost, rapid, and quantitative isothermal virus detection platforms that may have utility in HIV-1 viral load monitoring by novice users at POC and at-home settings. The digital RT-LAMP assay takes advantage of massively parallel micro-reactor chamber typically fabricated through microfluidics to create quantitative fluorescence readout from positive samples. A recent improvement of this technology replaces the necessity for fabrication of microchambers used in commercially available track-etched filter membranes with defined pore sizes. The densely packed pores serve as the micro reaction chambers wherein the RT-LAMP reactions take place in a quantitative manner. The evolution of florescence readout can be monitored by inexpensive complementary metal oxide semiconductor (CMOS) cameras or using a smartphone camera interface [140,142,152]. This method does not rely on expensive microfabrication or thermocyclers, while taking advantage of a smartphone as the optical reader, signal processor, and user interface. It produces nucleic acid amplification results comparable to quantitative PCR [153] with similar speeds, and, thus, has potential as a POC device for HIV self-monitoring. This sensing approach can also be further improved by performing blood filtration through the pores of the membrane while carrying out the RT-LAMP within the same pore walls [154].

Molecularly Imprinted Polymers (MIPs) integrated with various sensing technologies such as electrochemical, electroluminescence, fluorescence, cyclic voltammetry is emerging as a promising biodetection technology that is highly suitable as a POC or at-home virus detection tool [155,156,157,158]. MIPs are structural mimics of antibodies and other similar bioanalyte recognition elements. They are also referred to as plastic antibodies since they are formed from functional polymers, can capture analytes with high affinity, but are highly stable, easy to fabricate, and readily integrate into portable device schemes. Recently, MIPs-based electrochemical nanosensors were successfully used to detect SAR-CoV-2 and HIV through a POC diagnostic approach [159].

Among the widely popular LFA format testing devices with optical readout, a new class of Aggregation-Induced Emission (AIE) approach [160] is receiving fresh attention since it has interesting potentials for biodetection for POC use or home/self-monitoring purposes. This sensing approach offers several unique advantages, such as its reduced noise in the nonaggregated state, capability for a dominant optical emission/detection luminescence signal, resistance to photobleaching, and lack of blinking behavior, providing advantages over alternative florescence or luminescence-based optical sensors [160,161].

A compact and filter-free luminescence biosensor utilized Up-Conversion Nanoparticles (UCNP) as reporters to detect several classes of bioanalytes [129]. In this scheme, the optical excitation and detection were temporally modulated so that the luminescence readout is achieved after the excitation source is turned-off as the UCNPs can emit luminesce with time decay after pulse excitation. This clever solution resulted in the benefit of requiring neither expensive excitation and emission filters nor costly optical sensors, as the device could use a smartphone camera as the optical sensor. Furthermore, since there are typically no known luminescence sources in the biological samples, autofluorescence from non-target molecules were eliminated, resulting in a high signal-to-noise ratio detection. This type of an optical detection scheme is highly amenable for cost-effective POC use in HIV self-monitoring.

FET sensors have recently been used for the detection of SARS-CoV-2 manufactured by two main routes: (a) bottom-up fabrication, such as through the use of carbon nanotubes (CNT) [162,163] or graphene [164,165] or other “1D” materials as the sensing layer, and b) silicon-on-isolator (SOI) FET sensor technology. The principle of their operation relies on electrical interaction of the surface charges of these molecules (field effect) with the gate-layer of the FET, resulting in modulation of the current flow between the source and drain contacts of the device. Since these highly sensitive, multiplexable, and mass-producible sensors are fabricated by the conventional top-down CMOS technology, they are expected to be low cost and highly likely to be translated into commercial products for home/self-use purposes. Currently, one of the biggest limitations on achieving such devices is the requirement for a costly and highly complex detection instrument that can be inexpensively manufactured. Most of this type of sensing requires regulated power sources, sensitive electrical readout instruments (impedance analyzer, potentiostats, ammeters, voltmeters, lock-in-amplifiers, signal conditioners). Furthermore, these biosensors are also susceptible to the fundamental limitations imposed by the non-specific interaction issues observed in biologically-based ‘binders’ such as specificity limitations and cross reactivity potentials of antibodies, aptamers, or nucleic acid-based hybridization probes. Despite these limitations, CMOS-compatible FET devices offer many advantages such as scalability, multiplex detection, integration with on-chip sensor, signal processing, and readout circuitry to form highly cost-effective portable devices or system-on-chip (SOC) devices with multiple functionalities that can make them potentially valuable tools in the HIV detection field. They offer real-time and high-sensitivity detection potential in an integrated small form factor device that is highly suitable for novice/untrained users.

**Figure 8:**
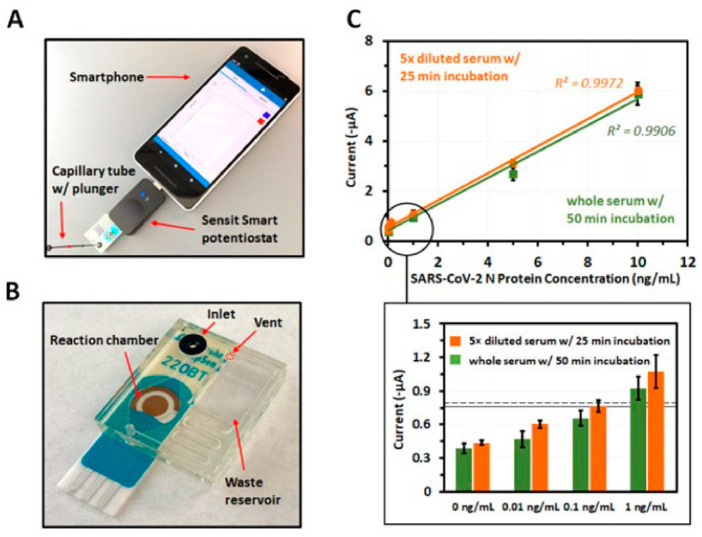
A modular smartphone-based readout scheme (**A**) for electrochemical detection of SARS-CoV-2 using a custom-made cartridge (**B**) that plugs into the USB port of the phone. The platform could detect viral N protein-generated amperometric signals from both whole and diluted serum samples reliably (**C**) at protein concentrations ranging from 0 to 1 ng/mL ((**C**), below) [166]. The figures reprinted with permission from ACS Sens. Copyright © 2021 American Chemical Society.

The use of magnetic/paramagnetic nanoparticles that are dually functionalized with a target detecting ‘binder’ (antibody, aptamer, nanobody) and an optically reporting signal generating enzyme (horse radish peroxidase, luciferase, alkaline phosphatase) offers interesting opportunities for HIV self-testing applications. For example, virus enrichment from undiluted serum via magnetic virus capture and subsequent electrical signal generation by horse radish peroxidase for SARS-CoV-2 detection in a small form factor device used a commercially available small potentiostat (Sensit Smart, Palmsens Inc, Houten, The Netherlands) that can be inserted into the USB port of a smartphone as seen in Figure 8 [166]. Utilization of these types of off-the-shelf available miniaturized electrical/electronic sensing devices with electrical, electrochemical output (voltammetry, amperometry, electrochemical impedance) capability that can simply be inserted into smartphones or a user’s standard laptop/desktop could find wide utility in POC HIV detection.

## 7. Discussion

This review of existing technologies describes HIV self-testing that can be conducted at home or through POC, similar to those authorized by the FDA for OTC/at-home use for COVID-19. There are several hurdles to overcome that extend beyond the technological aspects. For example, research innovations face a funding gap between early-stage technology development and commercialization that can be filled by government, or private venture, or philanthropic investments. For a technology to gain clinical adoption it must gain support [167] from various components of the healthcare system, including insurance/reimbursement coverage before it becomes established as part of the standard-of-care or part of routine infectious disease monitoring tool for the general public. Hence, there must be willingness to change current medical practices, insurance coverage, the habits of healthcare professionals, improvements in logistical access of the at-risk populations to testing devices, easing of approval processes of such devices by the regulatory agencies, and encouragement of adoption of those devices by the end users.

The goals of the NIH Rapid Acceleration of Diagnostics (RADx) Tech Program [168] is to accelerate the commercialization of promising and innovative POC and home-based tests as well as to improve laboratory-based tests, to detect SARS-CoV-2 directly. Although the products emerging from this program are potentially highly suitable for HIV self-testing at home or POC [169], the vast majority of COVID-19 detection is still performed using decades old RT-qPCR and LFA technologies. Concerningly, many of the newer technologies developed have been considered unsuitable for mass deployment. The underlying causes for this are multi-layered and intricate, ranging from technical issues such as large-scale manufacturability, robustness, reliability, and affordability to difficulties in regulatory approvals and raising funds for commercialization. Nevertheless, these activities currently require ‘self-testing’ with certified results that necessitate the involvement of healthcare professionals for reimbursement, to initiate a treatment, or switch the method of treatments, which diminishes their wide-scale adoption by the end users. Addressing these challenges is critical for the maturation and wide-scale adoption of POC testing-based diagnostic technologies for HIV self-testing.

As a result of the broad push towards tackling these challenges, it will become increasingly important that the requirements for POC/self-testing diagnostic devices are capable of handling multiple sample types and integrate downstream pre-processing workflow, while being self-contained, highly affordable, self-powered, and inexpensively manufactured. To guide these developments and to improve upon the WHO’s ASSURED criteria for optimal devices suitable for POC testing, ease of specimen collection and real-time connectivity properties were added to ASSURED, thus creating the REASSURED criteria [170]. The REASSURED criteria involve real-time connectivity (R), ease of specimen collection (E), affordable (A), sensitive (S), specific (S), user-friendly (U), rapid and robust (R), equipment free or simple (E), and deliverable to end-users (D). Especially after the raised awareness of the importance and acceptability of POC testing in the COVID-19 pandemic by the healthcare professionals, governments, and the general public, having access to such REASSURED devices and their adoption can provide instant actionable data for improved patient outcomes, better infectious disease transmission control and prevention strategies, and ultimately improved quality of life.

More than a decade ago, the biomedical framework “HIV treatment as prevention” began in earnest. It is a highly effective method of HIV prevention, wherein individuals living with HIV who are engaged in care can reduce their HIV viral load to levels undetectable by current laboratory testing standards and eliminate sexual HIV transmission risk to partners [171]. This evidence-based framework has led to public health campaigns promoting “undetectable equals untransmittable,” or “U=U”, to help reduce HIV stigma and promote HIV testing and treatment [172]. Nonetheless, fewer than half of MSM living with HIV are engaged in treatment care, and the U.S. continues to experience more than 35,000 HIV infections yearly [173], requiring combined biomedical and behavioral HIV prevention approaches to be inclusive of HIV-negative individuals.

Complementary care, such as home viral load testing, would help people living with HIV maintain viral suppression, improve overall health, and prevent HIV transmission. In a 2015 online survey of 11,863 U.S. MSM living with HIV, 83% endorsed using a home viral load test if one becomes available [174]. Black and Hispanic/Latino MSM were significantly more likely than White MSM to report suboptimal ART adherence but also more likely to endorse the viral load home test. Developing such a test for use in conjunction with HIV clinical care could turn the tide for individuals who use injectable ART and require verification of viral suppression in between clinical visits, for those who cannot maintain durable viral suppression (e.g., continuous undetectable viral load over a 12-month period), or those who disengage from care and are unable to maintain a consistent viral load monitoring schedule. Advances in biosensor-based HIV self-testing are needed more than ever to stem the tide of HIV, reduce HIV stigma, and reach key populations.

## Figures and Tables

**Table 1:** Summary of HIV self-tests: WHO Prequalified (WHO PQ).

	Product Name	Manufacturer	Specimen	Time to Result	Analyte	Sensitivity/Specificity (95% CI)	Regulatory Approval
*	Wondfo HIV Self-Test [41]	Guangzhou Wondfo Biotech Co	WB	15–30 min	HIV-1/2 Ab	95.8%/99.6%	WHO PQ,
*	CheckNOW HIV SELF TEST [42]	Abbott Rapid Diagnostics	WB	15–20 min	HIV-1/2 Ab	99.5%/98.5%	WHO PQ, CE marked
*	SURE CHECK^®^ HIV Self-Test [43]	Chembio Diagnostic Systems	WB	15–20 min	HIV-1/2 Ab	97%/100%	WHO PQ, FDA
*	Mylan HIV Self Test [44]	Atomo Diagnostics	WB	15–20 min	HIV-1/2 Ab	99.8%/99.8%	WHO PQ
*	INSTI^®^ HIV Self Test [45]	bioLytical Laboratories	WB	5 min	HIV-1/2 Ab	99.8%/99.5%	WHO PQ, CE marked
*	Exacto HIV self-test [46]	Biosynex	WB	10–20 min	HIV-1/2 Ab	100%/99.9%	CE marked
*	Autotest VIH^®^ [7]	AAZ	WB	15–20 min	HIV-1/2 Ab	99.86%/99.95%	WHO PQ, CE marked
*	Action! HIV Self-Test [47]	Chembio Diagnostic Systems	WB	10–20 min	HIV-1/2 Ab & p24 Ag	99.9%/99.9%	CE marked
*	AtomoRapid HIV Self-Test (3rd) [48]	Atomo Diagnostics	WB	15–20 min	HIV-1/2 Ab	99.6%/99.6%	CE marked
*	OraQuick^®^ HIV Self-Test [49]	OraSure Technologies	Oral fluid	20–40 min	HIV-1/2 Ab	99.4%/99.0%	WHO PQ, CE Marked
*	Saliteste HIV [50]	Ebram Product	WB	–	HIV-1/2 Ab	-/99.8%	
*	AWARE^TM^ HIV-1/2 OMT Oral HIV Self Test [51]	Calyple	Oral fluid	20–40 min	HIV-1/2 Ab	99.2%/99.9%	
**	HIV 1/2 STAT-PAK [52]	Chembio Diagnostic Systems	WB	15–20 min	HIV-1/2 Ab	99.52%/100%	WHO PQ, FDA
**	DPP HIV 1/2 Assay [53,54]	Chembio Diagnostic Systems	WB or oral fluid	30–45 min	HIV-1/2 Ab	100%/99.9%	WHO PQ, FDA CE marked
**	SURE CHECK^®^ HIV 1/2 Assay [54,55]	Chembio Diagnostic Systems	WB	15–20 min	HIV-1/2 Ab	99.8%/99.9%	WHO PQ, FDA
**	INSTI HIV-1/HIV-2 Antibody Test [56,57]	BioLytical Laboratories	WB	5 min	HIV-1/2 Ab	99.6%/99.3%	WHO PQ, FDA, CE Marked
**	Uni-Gold HIV [58]	Trinity Biotech Manufacturing	WB	10–12 min	HIV-1/2 Ab	99.76%/99.85%	WHO PQ
**	Genie Fast HIV 1/2 [50,59]	Bio-Rad	WB	10–30 min	HIV-1/2 Ab	100%/98.5%	WHO PQ, CE marked
**	MERISCREEN HIV 1-2 WB [60]	Meril Diagnostics	WB	20–30 min	HIV-1/2 Ab	99.4%/99.9%	WHO PQ, CE Marked
**	First Response HIV 1-2.O Card test (Version 2.0) [61]	Premier Medical Corporation Private Limited	WB	15–25 min	HIV-1/2 Ab	100%/100%	WHO PQ, CE Marked
**	ONE STEP Anti-HIV (1&2) Test [62,63]	InTec PRODUCTS	WB	15–20 min	HIV-1/2 Ab	100%/100%	WHO PQ, CE Marked
**	STANDARD Q HIV 1/2 Ab 3-Line Test [64]	SD Biosensor	WB	10–20 min	HIV-1/2 Ab	100%/99.3%	WHO PQ,
**	Bioline HIV-1/2 3.0 [65]	Abbott Diagnostics Korea	WB	10–20 min	HIV-1/2 Ab	100%/99.9%	WHO PQ,
**	Diagnostic kit for HIV (1 + 2) antibody (colloidal gold) V2 [66]	Shanghai Kehua Bioengineering	WB	15–25 min	HIV-1/2 Ab	100%/100%	WHO PQ,
**	OraQuick^®^ HIV 1/2 Advance Rapid Antibody Test [67]	OraSure Technologies	WB	20 min	HIV-1/2 Ab	99.1%/99.8%	WHO PQ, FDA
**	Rapid Test for Antibody to HIV (Colloidal Gold Device) [68]	Beijing Wantai Biological Pharmacy Enterprise	WB	15–20 min	HIV-1/2 Ab	100%/98.48%	WHO PQ,
**	ABON HIV 1/2/O Tri-Line Human Immunodeficiency Virus Rapid Test Device [69]	ABON Biopharm	WB	10–20 min	HIV-1/2 Ab	100%/99.7%	WHO PQ,
**	Determine HIV-1/2 [70]	Abbott Diagnostics Medical Co	WB	15–60 min	HIV-1/2 Ab	100%/98.93%	WHO PQ,
**	TrinScreen HIV [71]	Trinity Biotech Manufacturing	WB	10–12 min	HIV-1/2 Ab	100%/100%	WHO PQ,
**	Vikia HIV-1/2 [72]	bioMérieux	WB	30 min	HIV-1/2 Ab	99.86%/99.95%	CE marked
**	Reveal Rapid HIV-1 Antibody Test [73]	MedMira	WB	Immediate	HIV-1 Ab	99.8%/99.1%	FDA
**	iCARE One-Step HIV1&2 Whole Blood/Serum/Plasma Test [74]	JAL Innovation	WB	10 min	HIV-1/2 Ab		
-	Asanté™ HIV-1/2 Oral Fluid Rapid Test [75]	Sedia	Oral fluid	20 min	HIV1/2 Ab		
**	STANDARD Q HIV/Syphilis Combo Test [76]	SD Biosensor	WB	10–20 min	HIV-1/2 Ab, and syphilis	100%/99.9% (HIV) 98.8%/100% (Syphilis)	WHO PQ
**	First Response HIV1 + 2/Syphilis Combo Card Test [77]	Premier Medical Corporation Private Limited	WB	15–25 min	HIV-1/2 Ab, and syphilis	100%/99.5% (HIV-1/2) 99%/100% (Syphilis)	WHO PQ
**	Bioline HIV/Syphilis Duo [78]	Abbott Diagnostics Korea	WB	15–20 min	HIV-1/2 Ab, and syphilis	100%/99.5% (HIV-1/2) 87%/99.5% (Syphilis)	WHO PQ

* Indicates products intended for use by both professional healthcare and untrained lay users at POC setting. ** Indicates products intended for use by healthcare professionals at POC setting. -Indicates products under development or research purposes only.

**Table 2:** Self-sampling products accessible in the market currently.

	Product Name	Manufacturer	Specimen	Anolyte	Format	Regulatory Approval
*	Home Access HIV-1 Test System	Home Access Health Corp	WB	HIV-1 Ab	Dried blood spot	FDA
*	OraQuick In-Home HIV Test	OraSure Technologies	Oral fluid	HIV-1/2 Ab	Oral fluid	FDA
*	TASSO-M20	Tasso	WB		Dried blood spot	FDA, CE mark
*	OneDraw	DrawBridge Health	WB		Wet	FDA, CE marked
*	Capitainer qDBS	Capitainer	WB		Dried blood spot	FDA (Filed)
*	HemaPEN	Trajan Scientific and Medical	WB		Dried blood spot	
*	HemaSpot HF/SE/HD	Spot on Science	WB		Dried blood spot	
*	Mitra Blood Collection Device	Neoteryx	WB		Dried blood spot	
**	OraSure HIV-1 Oral Specimen Collection Device	OraSure Technologies	Oral fluid	HIV-1 Ab	Oral fluid	FDA
**	Haiim	Winnoz	WB		Wet	CE mark
*-*	HemaXis DB10	DBS System	WB		Dried blood spot	
*-*	Touch Activated Phlebotomy (TAP) 20C^TM^	Seventh Sense Biosystems	WB		Dried blood spot	
*-*	TASSO-SST	Tasso	WB		Wet	
*-*	PBS-1000	PreciHealth	WB		Wet	
*-*	Noviplex Cards	Shimadzu	WB		Dried plasma spot	
*-*	Book-Type Dried Plasma Spot Card		WB		Dried plasma spot	

* Indicates products intended for use by both professional healthcare and untrained lay users at POC setting. ** Indicates products intended for use by healthcare professionals at POC setting. -Indicates products under development or research purposes only.

## Data Availability

No new data were created or analyzed in this study. Data sharing is not applicable to this article.

## References

[1] Ollevier A., Aguiar G., Palomino M., Simpelaere I.S. (2020). How can technology support ageing in place in healthy older adults? A systematic review. Public Health Rev..

[2] Steehler K., Siegler A.J. (2019). Bringing HIV self-testing to scale in the United States: A review of challenges, potential solutions, and future opportunities. J. Clin. Microbiol..

[3] McGuire M., de Waal A., Karellis A., Janssen R., Engel N., Sampath R., Carmona S., Zwerling A.A., Suarez M.F., Pai N.P. (2021). HIV self-testing with digital supports as the new paradigm: A systematic review of global evidence (2010–2021). EClinicalMedicine.

[4] Ender P.T., Markson R.H., Suri A., Ruppert K., Padron N., Stoltzfus J.C., Berges V., Reed R. (2022). Rapid Implementation of a Telemedicine Program in a Ryan White-Funded HIV Clinic During a Global Pandemic. J. Acquir. Immune. Defic. Syndr..

[5] El-Nahal W.G., Shen N.M., Keruly J.C., Jones J.L., Fojo A.T., Lau B., Manabe Y.C., Moore R.D., Gebo K.A., Lesko C.R. (2022). Telemedicine and visit completion among people with HIV during the coronavirus disease 2019 pandemic compared with prepandemic. AIDS.

[6] Ochodo E.A., Olwanda E.E., Deeks J.J., Mallett S. (2022). Point-of-care viral load tests to detect high HIV viral load in people living with HIV/AIDS attending health facilities. Cochrane Database Syst. Rev..

[7] Branson B.M., Handsfield H.H., Lampe M.A., Janssen R.S., Taylor A.W., Lyss S.B., Clark J.E. (2006). Revised recommendations for HIV testing of adults, adolescents, and pregnant women in health-care settings. MMWR Recomm Rep..

[8] UNAIDS (2014). Fast-Track-Ending the AIDS Epidemic by 2030. https://www.unaids.org/en/resources/documents/2014/JC2686_WAD2014report.

[9] Group I.S.S., Lundgren J.D., Babiker A.G., Gordin F., Emery S., Grund B., Sharma S., Avihingsanon A., Cooper D.A., Fatkenheuer G. (2015). Initiation of Antiretroviral Therapy in Early Asymptomatic HIV Infection. N. Engl. J. Med..

[10] Centers for Disease Control and Prevention HIV Testing. https://www.cdc.gov/hiv/testing/index.html.

[11] Luo W., Sullivan V., Smith T., Peters P.J., Gay C., Westheimer E., Cohen S.E., Owen S.M., Masciotra S. (2019). Performance evaluation of the Bio-Rad Geenius HIV 1/2 supplemental assay. J. Clin. Virol..

[12] Marson K.G., Marlin R., Pham P., Cohen S.E., Jones D., Roemer M., Peters P.J., Haller B., Pilcher C.D. (2017). Real-world performance of the new US HIV testing algorithm in medical settings. J. Clin. Virol..

[13] HIV/AIDS. https://www.mayoclinic.org/diseases-conditions/hiv-aids/diagnosis-treatment/drc-20373531.

[14] House T.W. Fact Sheet: The Biden Administration Announces Americans Can Order Additional Free At-Home, Rapid COVID-19 Tests at COVIDTests.gov. https://www.whitehouse.gov/briefing-room/statements-releases/2022/05/17/fact-sheet-the-biden-administration-announces-americans-can-order-additional-free-at-home-rapid-covid-19-tests-at-covidtests-gov/.

[15] Weissleder R., Lee H., Ko J., Pittet M.J. (2020). COVID-19 diagnostics in context. Sci. Transl. Med..

[16] Perez S.M. (2022). Clusters of Rapid HIV Transmission Among Gay, Bisexual, and Other Men Who Have Sex with Men—United States, 2018–2021. MMWR. Morb. Mortal. Wkly. Rep..

[17] Ghosh S., Li N., Xiong Y., Ju Y.G., Rathslag M.P., Onal E.G., Falkiewicz E., Kohli M., Cunningham B.T. (2021). A compact photonic resonator absorption microscope for point of care digital resolution nucleic acid molecular diagnostics. Biomed. Opt. Express.

[18] Colasanti J.A., Armstrong W.S. (2019). Challenges of reaching 90–90–90 in the Southern United States. Curr. Opin. HIV AIDS.

[19] Deeks S.G., Lewin S.R., Havlir D.V. (2013). The end of AIDS: HIV infection as a chronic disease. Lancet.

[20] US Food & Drug Administration FDA in Brief: FDA Continues to Encourage Ongoing Education about the Benefits and Risks Associated with PrEP, Including Additional Steps to Help Reduce the Risk of Getting HIV. https://www.fda.gov/news-events/fda-brief/fda-brief-fda-continues-encourage-ongoing-education-about-benefits-and-risks-associated-prep.

[21] Beyrer C., McCormack S., Grulich A. (2022). Pre-Exposure Prophylaxis for HIV Infection as a Public Health Tool. J. Law Med. Ethics.

[22] Grispen J.E., Ronda G., Dinant G.J., de Vries N.K., van der Weijden T. (2011). To test or not to test: A cross-sectional survey of the psychosocial determinants of self-testing for cholesterol, glucose, and HIV. BMC Public Health.

[23] Pitasi M.A., Beer L., Cha S., Lyons S.J., Hernandez A.L., Prejean J., Valleroy L.A., Crim S.M., Trujillo L., Hardman D. (2021). Vital Signs: HIV Infection, Diagnosis, Treatment, and Prevention Among Gay, Bisexual, and Other Men Who Have Sex with Men—United States, 2010–2019. MMWR Morb. Mortal. Wkly Rep..

[24] Peabody R. Home HIV Testing (Self-Testing and Self-Sampling). https://www.aidsmap.com/about-hiv/home-hiv-testing-self-testing-and-self-sampling.

[25] Orasure Technologies OraQuick® In-Home HIV Test. https://oraquick.com/oraquick-in-home-hiv-test/.

[26] World Health Organization WHO List of Prequalified In Vitro Diagnostic Products. https://extranet.who.int/pqweb/vitro-diagnostics/vitro-diagnostics-lists.

[27] Banerjee R., Jaiswal A. (2018). Recent advances in nanoparticle-based lateral flow immunoassay as a point-of-care diagnostic tool for infectious agents and diseases. Analyst.

[28] Koczula K.M., Gallotta A. (2016). Lateral flow assays. Essays Biochem..

[29] Suarez-Pantaleon C., Wichers J., Abad-Somovilla A., van Amerongen A., Abad-Fuentes A. (2013). Development of an immunochromatographic assay based on carbon nanoparticles for the determination of the phytoregulator forchlorfenuron. Biosens. Bioelectron..

[30] Bahadir E.B. (2016). Lateral flow assays: Principles, designs and labels. Trends Anal. Chem..

[31] Yang W., Li X.-B., Liu G.-W., Zhang B.-B., Zhang Y., Kong T., Tang J.-J., Li D.-N., Wang Z. (2011). A colloidal gold probe-based silver enhancement immunochromatographic assay for the rapid detection of abrin-a. Biosens. Bioelectron..

[32] Panferov V.G., Safenkova I.V., Zherdev A.V., Dzantiev B.B. (2018). Post-assay growth of gold nanoparticles as a tool for highly sensitive lateral flow immunoassay. Application to the detection of potato virus X. Microchim. Acta.

[33] WHO Prequalification of In Vitro Diagnostics (2022). Product: Determine HIV-1/2. Prequalification of In Vitro Diagnostics Public Report.

[34] Wang Z., Zhi D., Zhao Y., Zhang H., Wang X., Ru Y., Li H. (2014). Lateral flow test strip based on colloidal selenium immunoassay for rapid detection of melamine in milk, milk powder, and animal feed. Int. J. Nanomed..

[35] Noguera P.S., Posthuma-Trumpie G.A., van Tuil M., van der Wal F.J., Boer A.d., Moers A.P.H.A., van Amerongen A. (2011). Carbon Nanoparticles as Detection Labels in Antibody Microarrays. Detection of Genes Encoding Virulence Factors in Shiga Toxin-Producing Escherichia coli. Anal. Chem..

[36] Workman S., Wells S.K., Pau C.P., Owen S.M., Dong X.F., LaBorde R., Granade T.C. (2009). Rapid detection of HIV-1 p24 antigen using magnetic immuno-chromatography (MICT). J. Virol. Methods.

[37] Deng X., Wang C., Gao Y., Li J., Wen W., Zhang X., Wang S. (2018). Applying strand displacement amplification to quantum dots-based fluorescent lateral flow assay strips for HIV-DNA detection. Biosens. Bioelectron..

[38] Fu X., Cheng Z., Yu J., Choo P., Chen L., Choo J. (2016). A SERS-based lateral flow assay biosensor for highly sensitive detection of HIV-1 DNA. Biosens. Bioelectron..

[39] Patel H.K., Ikpe S., Bronson M., Birhanu S., Abimiku A.l., Jahun I., Detorio M., Lupoli K., Yavo D., Bassey O.O. (2022). Performance of HIV rapid testing algorithm in Nigeria: Findings from a household-based Nigeria HIV/AIDS Indicator and Impact Survey (NAIIS). PLoS Glob. Public Health.

[40] Baveewo S., Kamya M.R., Mayanja-Kizza H., Fatch R., Bangsberg D.R., Coates T., Hahn J.A., Wanyenze R.K. (2012). Potential for false positive HIV test results with the serial rapid HIV testing algorithm. BMC Res. Notes.

[41] WHO Prequalification of In Vitro Diagnostics (2022). Product: Wondfo HIV Self-Test. Prequalification of In Vitro Diagnostics Public Report.

[42] WHO Prequalification of In Vitro Diagnostics (2022). Product: CheckNOW HIV SELF TEST. Prequalification of In Vitro Diagnostics Public Report.

[43] WHO Prequalification of In Vitro Diagnostics (2019). Product: SURE CHECK HIV Self-Test. Prequalification of In Vitro Diagnostics Public Report.

[44] WHO Prequalification of In Vitro Diagnostics (2021). Product: Mylan HIV Self Test. Prequalification of In Vitro Diagnostics Public Report.

[45] WHO Prequalification of In Vitro Diagnostics (2021). Product: INSTI HIV Self Test. Prequalification of In Vitro Diagnostics Public Report.

[46] Mossoro-Kpinde C.D., Bobossi C., Tonen-Wolyec S., Kalla G.C.M., Baguida-Bokia C., Sombot-Ndicki S., Gresenguet G., Mbopi-Keou F.X., Belec L. (2022). Analytical performances of Exacto((R)) HIV self-test in the Central African Republic. Pan. Afr. Med. J..

[47] (2020). UNAIDS. Understanding Fast-Track: Accelerating Action to End the Aids Epidemic by 2030. https://www.unaids.org/sites/default/files/media_asset/201506_JC2743_Understanding_FastTrack_en.pdf.

[48] Lippman S.A., Gilmore H.J., Lane T., Radebe O., Chen Y.H., Mlotshwa N., Maleke K., Manyuchi A.E., McIntyre J. (2018). Ability to use oral fluid and fingerstick HIV self-testing (HIVST) among South African MSM. PLoS ONE.

[49] WHO Prequalification of In Vitro Diagnostics (2022). Product: OraQuick HIV Self-Test. Prequalification of In Vitro Diagnostics Public Report.

[50] Kosack C.S., Page A.L., Beelaert G., Benson T., Savane A., Ng’ang’a A., Andre B., Zahinda J.B., Shanks L., Fransen K. (2017). Towards more accurate HIV testing in sub-Saharan Africa: A multi-site evaluation of HIV RDTs and risk factors for false positives. J. Int. AIDS Soc..

[51] Alemnji G.A., Ngulefac G.A., Ndumbe P.M., Asonganyi T. (2009). Field evaluation of Calypte’s AWARE blood serum plasma (BSP) and oral mucosal transudate (OMT) rapid tests for detecting antibodies to HIV-1 and 2 in plasma and oral fluid. Open AIDS J..

[52] WHO Prequalification of In Vitro Diagnostics (2012). Product: HIV 1/2 STAT-PAK®. Prequalification of In Vitro Diagnostics Public Report.

[53] WHO Prequalification of In Vitro Diagnostics (2018). Product: DPP® HIV 1/2 Assay. Prequalification of In Vitro Diagnostics Public Report.

[54] Jaspard M., Le Moal G., Saberan-Roncato M., Plainchamp D., Langlois A., Camps P., Guigon A., Hocqueloux L., Prazuck T. (2014). Finger-stick whole blood HIV-1/-2 home-use tests are more sensitive than oral fluid-based in-home HIV tests. PLoS ONE.

[55] WHO Prequalification of In Vitro Diagnostics (2020). Product: SURE CHECK HIV 1/2 Assay. Prequalification of In Vitro Diagnostics Public Report.

[56] Mayaphi S.H., Martin D.J., Quinn T.C., Stoltz A.C. (2019). Field performance of the INSTI HIV-1/-2 antibody test in two South African antenatal clinics. J. Med. Virol..

[57] WHO Prequalification of In Vitro Diagnostics (2019). Product: INSTI HIV-1/HIV-2 Antibody Test. Prequalification of In Vitro Diagnostics Public Report.

[58] WHO Prequalification of In Vitro Diagnostics (2021). Product: Uni-Gold HIV. Prequalification of In Vitro Diagnostics Public Report.

[59] WHO Prequalification of In Vitro Diagnostics (2017). Product: Genie™ Fast HIV 1/2. Prequalification of In Vitro Diagnostics Public Report.

[60] WHO Prequalification of In Vitro Diagnostics (2020). Product: MERISCREEN HIV 1-2 WB. Prequalification of In Vitro Diagnostics Public Report.

[61] Boadu R., Darko G., Nortey P., Akweongo P., Sarfo B. (2016). Assessing the sensitivity and specificity of First Response HIV-1-2 test kit with whole blood and serum samples: A cross-sectional study. AIDS Res. Ther..

[62] WHO Prequalification of In Vitro Diagnostics (2020). Product: ONE STEP Anti-HIV (1&2) Test. Prequalification of In Vitro Diagnostics Public Report.

[63] Turbe V., Herbst C., Mngomezulu T., Meshkinfamfard S., Dlamini N., Mhlongo T., Smit T., Cherepanova V., Shimada K., Budd J. (2021). Deep learning of HIV field-based rapid tests. Nat. Med..

[64] WHO Prequalification of In Vitro Diagnostics (2020). Product: STANDARD Q HIV 1/2 Ab 3-Line Test. Prequalification of In Vitro Diagnostics Public Report.

[65] WHO Prequalification of In Vitro Diagnostics (2020). Product: Bioline HIV 1/2 3.0. Prequalification of In Vitro Diagnostics Public Report.

[66] WHO Prequalification of In Vitro Diagnostics (2016). Product: Diagnostic kit for HIV (1+2) antibody (colloidal gold) V2. Prequalification of In Vitro Diagnostics Public Report.

[67] Guillon G., Yearwood G., Snipes C., Boschi D., Reed M.R. (2018). Human anti-HIV IgM detection by the OraQuick ADVANCE(R) Rapid HIV 1/2 Antibody Test. PeerJ.

[68] WHO Prequalification of In Vitro Diagnostics (2020). Product: Rapid Test for Antibody to Human Immunodeficiency Virus (HIV) (Colloidal Gold Device). Prequalification of In Vitro Diagnostics Public Report.

[69] WHO Prequalification of In Vitro Diagnostics (2019). Product: ABON HIV 1/2/O Tri-Line Human Immunodeficiency Virus Rapid Test Device. Prequalification of In Vitro Diagnostics Public Report.

[70] van den Berk G.E., Frissen P.H., Regez R.M., Rietra P.J. (2003). Evaluation of the rapid immunoassay determine HIV 1/2 for detection of antibodies to human immunodeficiency virus types 1 and 2. J. Clin. Microbiol..

[71] WHO Prequalification of In Vitro Diagnostics (2022). Product: TrinScreen HIV. Prequalification of In Vitro Diagnostics Public Report.

[72] da Motta L.R., Vanni A.C., Kato S.K., Borges L.G., Sperhacke R.D., Ribeiro R.M., Inocencio L.A., The HIV Rapid Test Evaluation Group (2013). Evaluation of five simple rapid HIV assays for potential use in the Brazilian national HIV testing algorithm. J. Virol. Methods.

[73] Rossetti R., Smith T., Luo W., Masciotra S. (2020). Performance evaluation of the MedMira reveal G4 LAB S/P and POC HIV antibody rapid screening tests using plasma and whole blood specimens. J. Clin. Virol..

[74] Wechsberg W.M., van der Horst C., Ndirangu J., Doherty I.A., Kline T., Browne F.A., Belus J.M., Nance R., Zule W.A. (2017). Seek, test, treat: Substance-using women in the HIV treatment cascade in South Africa. Addict. Sci. Clin. Pract..

[75] Majam M., Rhagnath N., Msolomba V., Singh L., Urdea M.S., Lalla-Edward S.T. (2021). Usability and Clinical Performance Characteristics of the Asante HIV1/2 Test by Trained Users in Two African Sites. Diagnostics.

[76] WHO Prequalification of In Vitro Diagnostics (2020). Product: STANDARD Q HIV/Syphilis Combo Test. Prequalification of In Vitro Diagnostics Public Report.

[77] WHO Prequalification of In Vitro Diagnostics (2020). Product: First Response HIV1+2/Syphilis Combo Card Test. Prequalification of In Vitro Diagnostics Public Report.

[78] WHO Prequalification of In Vitro Diagnostics (2022). Product: Bioline HIV/Syphilis Duo. Prequalification of In Vitro Diagnostics Public Report.

[79] Gruner N., Stambouli O., Ross R.S. (2015). Dried blood spots--preparing and processing for use in immunoassays and in molecular techniques. J. Vis. Exp..

[80] Lim M.D. (2018). Dried Blood Spots for Global Health Diagnostics and Surveillance: Opportunities and Challenges. Am. J. Trop Med. Hyg..

[81] Delahaye L., Veenhof H., Koch B.C.P., Alffenaar J.C., Linden R., Stove C. (2021). Alternative Sampling Devices to Collect Dried Blood Microsamples: State-of-the-Art. Drug Monit..

[82] Marchand A., Roulland I., Semence F., Beck O., Ericsson M. (2021). Use of Quantitative Dried Blood Spots to Evaluate the Post-Vaccination Level of Neutralizing Antibodies against SARS-CoV-2. Life.

[83] Leuthold L.A., Heudi O., Deglon J., Raccuglia M., Augsburger M., Picard F., Kretz O., Thomas A. (2015). New microfluidic-based sampling procedure for overcoming the hematocrit problem associated with dried blood spot analysis. Anal. Chem..

[84] Zasada A.A., Zacharczuk K., Woznica K., Glowka M., Ziolkowski R., Malinowska E. (2020). The influence of a swab type on the results of point-of-care tests. AMB Express.

[85] Microbiology R. Flocked Swabs Proven Superior in Sample Uptake and Release. https://www.rapidmicrobiology.com/news/flocked-swabs-proven-superior-in-sample-uptake-and-release.

[86] Chen G.D., Alberts C.J., Rodriguez W., Toner M. (2010). Concentration and Purification of Human Immunodeficiency Virus Type 1 Virions by Microfluidic Separation of Superparamagnetic Nanoparticles. Anal. Chem..

[87] Lien K.-Y., Lin J.-L., Liu C.-Y., Lei H.-Y., Lee G.-B. (2007). Purification and enrichment of virus samples utilizing magnetic beads on a microfluidic system. Lab Chip.

[88] Wang S., Esfahani M., Gurkan U.A., Inci F., Kuritzkes D.R., Demirci U. (2012). Efficient on-chip isolation of HIV subtypes. Lab Chip.

[89] Wang S., Xu F., Demirci U. (2010). Advances in developing HIV-1 viral load assays for resource-limited settings. Biotechnol. Adv..

[90] Okoye A.A., Picker L.J. (2013). CD4(+) T-cell depletion in HIV infection: Mechanisms of immunological failure. Immunol. Rev..

[91] Thorsen T., Maerkl S.J., Quake S.R. (2002). Microfluidic large-scale integration. Science.

[92] Wang S., Tasoglu S., Chen P.Z., Chen M., Akbas R., Wach S., Ozdemir C.I., Gurkan U.A., Giguel F.F., Kuritzkes D.R. (2014). Micro-a-fluidics ELISA for Rapid CD4 Cell Count at the Point-of-Care. Sci. Rep..

[93] World Health Orgainzation Consolidated Guidelines On the Use of Antiretroviral Drugs for Treating and Preventing HIV Infection: Recommendations for a Public Health Approach. https://www.who.int/publications/i/item/9789241549684.

[94] Teeparuksapun K., Hedström M., Wong E.Y., Tang S., Hewlett I.K., Mattiasson B. (2010). Ultrasensitive Detection of HIV-1 p24 Antigen Using Nanofunctionalized Surfaces in a Capacitive Immunosensor. Anal. Chem..

[95] Bangalee A., Bhoora S., Punchoo R. (2021). Evaluation of serological assays for the diagnosis of HIV infection in adults. S. Afr. Fam. Pract. (2004).

[96] Sickinger E., Stieler M., Kaufman B., Kapprell H.P., West D., Sandridge A., Devare S., Schochetman G., Hunt J.C., Daghfal D. (2004). Multicenter evaluation of a new, automated enzyme-linked immunoassay for detection of human immunodeficiency virus-specific antibodies and antigen. J. Clin. Microbiol..

[97] Du M., Li N., Mao G., Liu Y., Wang X., Tian S., Hu Q., Ji X., Liu Y., He Z. (2019). Self-assembled fluorescent Ce(Ⅲ) coordination polymer as ratiometric probe for HIV antigen detection. Anal. Chim. Acta.

[98] Kurdekar A.D., Avinash Chunduri L.A., Manohar C.S., Haleyurgirisetty M.K., Hewlett I.K., Venkataramaniah K. (2018). Streptavidin-conjugated gold nanoclusters as ultrasensitive fluorescent sensors for early diagnosis of HIV infection. Sci. Adv..

[99] Li F., Zheng Y., Yang M., Zhang Y., Pu Q. (2022). A triple-layer microchannel array chip with micro through-holes for smartphone based point-of-care testing of biomarker. Sens. Actuators B Chem..

[100] Ng E., Yao C., Shultz T.O., Ross-Howe S., Wang S.X. (2019). Magneto-nanosensor smartphone platform for the detection of HIV and leukocytosis at point-of-care. Nanomedicine.

[101] Choi J., Gani A.W., Bechstein D.J.B., Lee J.R., Utz P.J., Wang S.X. (2016). Portable, one-step, and rapid GMR biosensor platform with smartphone interface. Biosens. Bioelectron..

[102] Che C., Li N., Long K.D., Aguirre M.A., Canady T.D., Huang Q., Demirci U., Cunningham B.T. (2019). Activate capture and digital counting (AC + DC) assay for protein biomarker detection integrated with a self-powered microfluidic cartridge. Lab Chip.

[103] Canady T.D., Li N., Smith L.D., Lu Y., Kohli M., Smith A.M., Cunningham B.T. (2019). Digital-resolution detection of microRNA with single-base selectivity by photonic resonator absorption microscopy. Proc. Natl. Acad. Sci. USA.

[104] Zhao B., Che C., Wang W., Li N., Cunningham B.T. (2021). Single-step, wash-free digital immunoassay for rapid quantitative analysis of serological antibody against SARS-CoV-2 by photonic resonator absorption microscopy. Talanta.

[105] Zhao B., Wang W., Li N., Garcia-Lezana T., Che C., Wang X., Losic B., Villanueva A., Cunningham B.T. (2022). Digital-resolution and highly sensitive detection of multiple exosomal small RNAs by DNA toehold probe-based photonic resonator absorption microscopy. Talanta.

[106] Xiong Y., Li N., Che C., Wang W., Barya P., Liu W., Liu L., Wang X., Wu S., Hu H. (2022). Microscopies Enabled by Photonic Metamaterials. Sensors.

[107] Centers for Disease Control and Prevention HIV Testing: Understanding the HIV Window Period. https://www.cdc.gov/hiv/basics/hiv-testing/hiv-window-period.html.

[108] Dobaño C., Vidal M., Santano R., Jiménez A., Chi J., Barrios D., Ruiz-Olalla G., Melero N.R., Carolis C., Parras D. (2020). Highly sensitive and specific multiplex antibody assays to quantify immunoglobulins M, A and G against SARS-CoV-2 antigens. bioRxiv.

[109] Alexander T.S. (2016). Human Immunodeficiency Virus Diagnostic Testing: 30 Years of Evolution. Clin. Vaccine Immunol..

[110] Daskalakis D. (2011). HIV diagnostic testing: Evolving technology and testing strategies. Top Antivir. Med..

[111] Liu J., Geng Z., Fan Z., Liu J., Chen H. (2019). Point-of-care testing based on smartphone: The current state-of-the-art (2017-2018). Biosens. Bioelectron..

[112] Cunningham B., Canady T., Zhao B., Ghosh S., Li N., Huang Q., Xiong Y., Fried G., Kohli M., Demirci U. (2021). Photonic Metamaterial Surfaces for Digital Resolution Biosensor Microscopies Using Enhanced Absorption, Scattering, and Emission.

[113] Guo T., Patnaik R., Kuhlmann K., Rai A.J., Sia S.K. (2015). Smartphone dongle for simultaneous measurement of hemoglobin concentration and detection of HIV antibodies. Lab Chip.

[114] Laksanasopin T., Guo T.W., Nayak S., Sridhara A.A., Xie S., Olowookere O.O., Cadinu P., Meng F., Chee N.H., Kim J. (2015). A smartphone dongle for diagnosis of infectious diseases at the point of care. Sci. Transl. Med..

[115] Mudanyali O., Dimitrov S., Sikora U., Padmanabhan S., Navruz I., Ozcan A. (2012). Integrated rapid-diagnostic-test reader platform on a cellphone. Lab Chip.

[116] Garg N., Boyle D., Randall A., Teng A., Pablo J., Liang X., Camerini D., Lee A.P. (2019). Rapid immunodiagnostics of multiple viral infections in an acoustic microstreaming device with serum and saliva samples. Lab Chip.

[117] Oliveira B.B., Veigas B., Baptista P.V. (2021). Isothermal Amplification of Nucleic Acids: The Race for the Next “Gold Standard”. Front. Sens..

[118] Liang Y., Li L., Shui J., Hu F., Wang H., Xia Y., Cai W., Tang S. (2020). Reduction of anti-HIV antibody responses in subjects receiving antiretroviral therapy during chronic HIV-1 infection. J. Clin. Virol..

[119] Weld E.D. (2020). Limits of Detection and Limits of Infection: Quantitative HIV Measurement in the Era of U = U. J. Appl. Lab. Med..

[120] Damhorst G.L., Duarte-Guevara C., Chen W., Ghonge T., Cunningham B.T., Bashir R. (2015). Smartphone-Imaged HIV-1 Reverse-Transcription Loop-Mediated Isothermal Amplification (RT-LAMP) on a Chip from Whole Blood. Engineering.

[121] Jankelow A.M., Lee H., Wang W., Hoang T.-H., Bacon A., Sun F., Chae S., Kindratenko V., Koprowski K., Stavins R.A. (2022). Smartphone clip-on instrument and microfluidic processor for rapid sample-to-answer detection of Zika virus in whole blood using spatial RT-LAMP. Analyst.

[122] Mauk M., Song J., Bau H.H., Gross R., Bushman F.D., Collman R.G., Liu C. (2017). Miniaturized devices for point of care molecular detection of HIV. Lab Chip.

[123] Das D., Lin C.-W., Chuang H.-S. (2022). LAMP-Based Point-of-Care Biosensors for Rapid Pathogen Detection. Biosensors.

[124] Hosaka N., Ndembi N., Ishizaki A., Kageyama S., Numazaki K., Ichimura H. (2009). Rapid detection of human immunodeficiency virus type 1 group M by a reverse transcription-loop-mediated isothermal amplification assay. J. Virol. Methods.

[125] Liu T., Choi G., Tang Z., Kshirsagar A., Politza A.J., Guan W. (2022). Fingerpick Blood-Based Nucleic Acid Testing on A USB Interfaced Device towards HIV self-testing. Biosens. Bioelectron..

[126] Myers F.B., Henrikson R.H., Bone J., Lee L.P. (2013). A Handheld Point-of-Care Genomic Diagnostic System. PLoS ONE.

[127] Curtis K.A., Rudolph D.L., Nejad I., Singleton J., Beddoe A., Weigl B., LaBarre P., Owen S.M. (2012). Isothermal amplification using a chemical heating device for point-of-care detection of HIV-1. PLoS ONE.

[128] Kim D., Wei Q., Kim D.H., Tseng D., Zhang J., Pan E., Garner O., Ozcan A., Di Carlo D. (2018). Enzyme-Free Nucleic Acid Amplification Assay Using a Cellphone-Based Well Plate Fluorescence Reader. Anal. Chem..

[129] Huang C.-H., Park Y.I., Lin H.-Y., Pathania D., Park K.S., Avila-Wallace M., Castro C.M., Weissleder R., Lee H. (2019). Compact and Filter-Free Luminescence Biosensor for Mobile In Vitro Diagnoses. ACS Nano.

[130] Tarim E.A., Karakuzu B., Oksuz C., Sarigil O., Kizilkaya M., Al-Ruweidi M., Yalcin H.C., Ozcivici E., Tekin H.C. (2021). Microfluidic-based virus detection methods for respiratory diseases. Emergent Mater..

[131] Poghossian A., Jablonski M., Molinnus D., Wege C., Schöning M.J. (2020). Field-Effect Sensors for Virus Detection: From Ebola to SARS-CoV-2 and Plant Viral Enhancers. Front. Plant Sci..

[132] Syahir A., Usui K., Tomizaki K.Y., Kajikawa K., Mihara H. (2015). Label and Label-Free Detection Techniques for Protein Microarrays. Microarrays.

[133] Cunningham B.T., Laing L.G. (2008). Advantages and application of label-free detection assays in drug screening. Expert Opin. Drug Discov..

[134] Rapp B.E., Gruhl F.J., Länge K. (2010). Biosensors with label-free detection designed for diagnostic applications. Anal. Bioanal. Chem..

[135] Li N., Wang X., Tibbs J., Che C., Peinetti A.S., Zhao B., Liu L., Barya P., Cooper L., Rong L. (2022). Label-Free Digital Detection of Intact Virions by Enhanced Scattering Microscopy. J. Am. Chem. Soc..

[136] Inci F., Tokel O., Wang S., Gurkan U.A., Tasoglu S., Kuritzkes D.R., Demirci U. (2013). Nanoplasmonic Quantitative Detection of Intact Viruses from Unprocessed Whole Blood. ACS Nano.

[137] Chauhan N., Xiong Y., Ren S., Dwivedy A., Magazine N., Zhou L., Jin X., Zhang T., Cunningham B.T., Yao S. (2022). Net-Shaped DNA Nanostructures Designed for Rapid/Sensitive Detection and Potential Inhibition of the SARS-CoV-2 Virus. J. Am. Chem. Soc..

[138] Li N., Canady T.D., Huang Q., Wang X., Fried G.A., Cunningham B.T. (2021). Photonic resonator interferometric scattering microscopy. Nat. Commun..

[139] Huang C., Wang Y., Li X., Ren L., Zhao J., Hu Y., Zhang L., Fan G., Xu J., Gu X. (2020). Clinical features of patients infected with 2019 novel coronavirus in Wuhan, China. Lancet.

[140] Ding X., Mauk M.G., Yin K., Kadimisetty K., Liu C. (2019). Interfacing Pathogen Detection with Smartphones for Point-of-Care Applications. Anal. Chem..

[141] Malekjahani A., Sindhwani S., Syed A.M., Chan W.C.W. (2019). Engineering Steps for Mobile Point-of-Care Diagnostic Devices. Acc. Chem. Res..

[142] Xiao M., Tian F., Liu X., Zhou Q., Pan J., Luo Z., Yang M., Yi C. (2022). Virus Detection: From State-of-the-Art Laboratories to Smartphone-Based Point-of-Care Testing. Adv. Sci..

[143] Zhang Z., Ma P., Ahmed R., Wang J., Akin D., Soto F., Liu B.-F., Li P., Demirci U. (2022). Advanced Point-of-Care Testing Technologies for Human Acute Respiratory Virus Detection. Adv. Mater..

[144] Eissa S. (2022). Diagnostic biosensors for coronaviruses and recent developments. Adv. Biosens. Virus Detect..

[145] Renzoni A., Perez F., Ngo Nsoga M.T., Yerly S., Boehm E., Gayet-Ageron A., Kaiser L., Schibler M. (2021). Analytical Evaluation of Visby Medical RT-PCR Portable Device for Rapid Detection of SARS-CoV-2. Diagnostics.

[146] Donato L.J., Trivedi V.A., Stransky A.M., Misra A., Pritt B.S., Binnicker M.J., Karon B.S. (2021). Evaluation of the Cue Health point-of-care COVID-19 (SARS-CoV-2 nucleic acid amplification) test at a community drive through collection center. Diagn Microbiol. Infect. Dis..

[147] Iwamoto T., Sonobe T., Hayashi K. (2003). Loop-mediated isothermal amplification for direct detection of Mycobacterium tuberculosis complex, M. avium, and M. intracellulare in sputum samples. J. Clin. Microbiol..

[148] Compton J. (1991). Nucleic acid sequence-based amplification. Nature.

[149] Lillis L., Lehman D.A., Siverson J.B., Weis J., Cantera J., Parker M., Piepenburg O., Overbaugh J., Boyle D.S. (2016). Cross-subtype detection of HIV-1 using reverse transcription and recombinase polymerase amplification. J. Virol. Methods.

[150] Mota D.S., Guimarães J.M., Gandarilla A.M.D., Filho J.C.B.S., Brito W.R., Mariúba L.A.M. (2022). Recombinase polymerase amplification in the molecular diagnosis of microbiological targets and its applications. Can. J. Microbiol..

[151] Sun B., Shen F., McCalla S.E., Kreutz J.E., Karymov M.A., Ismagilov R.F. (2013). Mechanistic evaluation of the pros and cons of digital RT-LAMP for HIV-1 viral load quantification on a microfluidic device and improved efficiency via a two-step digital protocol. Anal. Chem..

[152] Rodriguez-Manzano J., Karymov M.A., Begolo S., Selck D.A., Zhukov D.V., Jue E., Ismagilov R.F. (2016). Reading Out Single-Molecule Digital RNA and DNA Isothermal Amplification in Nanoliter Volumes with Unmodified Camera Phones. ACS Nano.

[153] Lin X., Huang X., Urmann K., Xie X., Hoffmann M.R. (2019). Digital Loop-Mediated Isothermal Amplification on a Commercial Membrane. ACS Sens.

[154] Schlappi T.S., McCalla S.E., Schoepp N.G., Ismagilov R.F. (2016). Flow-through Capture and in Situ Amplification Can Enable Rapid Detection of a Few Single Molecules of Nucleic Acids from Several Milliliters of Solution. Anal. Chem..

[155] Amouzadeh Tabrizi M., Fernández-Blázquez J.P., Medina D.M., Acedo P. (2022). An ultrasensitive molecularly imprinted polymer-based electrochemical sensor for the determination of SARS-CoV-2-RBD by using macroporous gold screen-printed electrode. Biosens. Bioelectron..

[156] Ayankojo A.G., Boroznjak R., Reut J., Öpik A., Syritski V. (2022). Molecularly imprinted polymer based electrochemical sensor for quantitative detection of SARS-CoV-2 spike protein. Sens. Actuators B Chem..

[157] Ratautaite V., Boguzaite R., Brazys E., Ramanaviciene A., Ciplys E., Juozapaitis M., Slibinskas R., Bechelany M., Ramanavicius A. (2022). Molecularly imprinted polypyrrole based sensor for the detection of SARS-CoV-2 spike glycoprotein. Electrochim. Acta.

[158] Singhal A., Parihar A., Kumar N., Khan R. (2022). High throughput molecularly imprinted polymers based electrochemical nanosensors for point-of-care diagnostics of COVID-19. Mater. Lett..

[159] Ma Y., Shen X.L., Zeng Q., Wang H.S., Wang L.S. (2017). A multi-walled carbon nanotubes based molecularly imprinted polymers electrochemical sensor for the sensitive determination of HIV-p24. Talanta.

[160] Liu Z., Meng T., Tang X., Tian R., Guan W. (2021). The Promise of Aggregation-Induced Emission Luminogens for Detecting COVID-19. Front. Immunol..

[161] Li J., Fan Y.-Y., Wen J., Zhang J., Zhang Z.-Q. (2022). Metal-Enhanced Aggregation-Induced Emission Strategy for the HIV-I RNA-Binding Ligand Assay. Anal. Chem..

[162] Thanihaichelvan M., Surendran S.N., Kumanan T., Sutharsini U., Ravirajan P., Valluvan R., Tharsika T. (2022). Selective and electronic detection of COVID-19 (Coronavirus) using carbon nanotube field effect transistor-based biosensor: A proof-of-concept study. Mater. Today Proc..

[163] Shao W., Shurin M.R., Wheeler S.E., He X., Star A. (2021). Rapid Detection of SARS-CoV-2 Antigens Using High-Purity Semiconducting Single-Walled Carbon Nanotube-Based Field-Effect Transistors. ACS Appl. Mater. Interfaces.

[164] Seo G., Lee G., Kim M.J., Baek S.-H., Choi M., Ku K.B., Lee C.-S., Jun S., Park D., Kim H.G. (2020). Rapid Detection of COVID-19 Causative Virus (SARS-CoV-2) in Human Nasopharyngeal Swab Specimens Using Field-Effect Transistor-Based Biosensor. ACS Nano.

[165] Ali M.A., Hu C., Jahan S., Yuan B., Saleh M.S., Ju E., Gao S.-J., Panat R. (2021). Sensing of COVID-19 Antibodies in Seconds via Aerosol Jet Nanoprinted Reduced-Graphene-Oxide-Coated 3D Electrodes. Adv. Mater..

[166] Li J., Lillehoj P.B. (2021). Microfluidic Magneto Immunosensor for Rapid, High Sensitivity Measurements of SARS-CoV-2 Nucleocapsid Protein in Serum. ACS Sens..

[167] Boppart S.A., Richards-Kortum R. (2014). Point-of-care and point-of-procedure optical imaging technologies for primary care and global health. Sci. Transl. Med..

[168] RADx Programs. https://www.nibib.nih.gov/covid-19/radx-tech-program.

[169] Robinson M., Gaydos C., Van Der Pol B., McFall S., Hsieh Y.-H., Clarke W., Murphy R.L., Widdice L.E., Hirschhorn L.R., Rothman R. (2021). The Clinical Review Committee: Impact of the Development of In Vitro Diagnostic Tests for SARS-CoV-2 Within RADx Tech. IEEE Open J. Eng. Med. Biol..

[170] Land K.J., Boeras D.I., Chen X.-S., Ramsay A.R., Peeling R.W. (2019). REASSURED diagnostics to inform disease control strategies, strengthen health systems and improve patient outcomes. Nat Microbiol.

[171] Cohen M.S., Chen Y.Q., McCauley M., Gamble T., Hosseinipour M.C., Kumarasamy N., Hakim J.G., Kumwenda J., Grinsztejn B., Pilotto J.H. (2011). Prevention of HIV-1 infection with early antiretroviral therapy. N. Engl. J. Med..

[172] Okoli C., Van de Velde N., Richman B., Allan B., Castellanos E., Young B., Brough G., Eremin A., Corbelli G.M., Mc Britton M. (2021). Undetectable equals untransmittable (U = U): Awareness and associations with health outcomes among people living with HIV in 25 countries. Sex. Transm. Infect..

[173] Center for Disease Control and Protection HIV and Gay and Bisexual Men: HIV Diagnoses. https://www.cdc.gov/hiv/group/msm/msm-content/diagnoses.html.

[174] Hirshfield S., Downing M.J., Chiasson M.A., Houang S.T., Yoon I.S., Teran R. Would HIV-positive MSM use a home viral load test?. Proceedings of the International Association of Providers of AIDS Care.

